# Material symmetry recognition and property prediction accomplished by crystal capsule representation

**DOI:** 10.1038/s41467-023-40756-2

**Published:** 2023-08-25

**Authors:** Chao Liang, Yilimiranmu Rouzhahong, Caiyuan Ye, Chong Li, Biao Wang, Huashan Li

**Affiliations:** 1 School of Physics, Sun Yat-Sen University, Guangzhou, China; 2 Guangdong Provincial Key Laboratory of Magnetoelectric Physics and Devices, School of Physics, Sun Yat-sen University, Guangzhou, China; 3 Center for Neutron Science and Technology, School of Physics, Sun Yat-sen University, Guangzhou, China

**Keywords:** Atomistic models, Electronic properties and materials

## Abstract

Learning the global crystal symmetry and interpreting the equivariant information is crucial for accurately predicting material properties, yet remains to be fully accomplished by existing algorithms based on convolution networks. To overcome this challenge, here we develop a machine learning (ML) model, named symmetry-enhanced equivariance network (SEN), to build material representation with joint structure-chemical patterns, to encode important clusters embedded in the crystal structure, and to learn pattern equivariance in different scales via capsule transformers. Quantitative analyses of the intermediate matrices demonstrate that the intrinsic crystal symmetries and interactions between clusters have been exactly perceived by the SEN model and critically affect the prediction performances by reducing effective feature space. The mean absolute errors (MAEs) of 0.181 eV and 0.0161 eV/atom are obtained for predicting bandgap and formation energy in the MatBench dataset. The general and interpretable SEN model reveals the potential to design ML models by implicitly encoding feature relationship based on physical mechanisms.

## Abstract (web summary)

Learning global crystal symmetry and interpreting equivariance are crucial for developing ML model to predict electronic properties. Here authors propose a symmetry-enhanced model to simulate cluster interactions and to predict materials properties.

## Introduction

Machine learning (ML) approaches based on the statistical mechanisms have recently been employed in material science to accomplish accurate property prediction and inverse design^1–7^. So far, state-of-the-art deep learning (DL) models have successfully described simple atomic correlations as well as crystal spatial patterns, and thus offered predictions of electronic, mechanical and optical properties^8–10^. The perception of intrinsic symmetry is crucial for accurately and extrapolatively predicting material properties, as pointed out by Grisoni and Schneider et al.^11^, because it governs the relative atomic energy level distributions and affects the intensity of orbital hybridizations^12,13^. The symmetries of a crystal material are represented by the relevant space group, which is defined as the set of all coordinate transformations that map the equilibrium positions of an infinite crystalline solid into itself^14^. The space groups of crystal materials are sub-groups of the Euclidean group. From the ML perspective, crystal symmetries are perceived as invariance and equivariance of materials, which should be automatically identified via recognizing the equivalent microscopic sub-structures with all characteristic scales^15–17^.

**Fig. 1:**
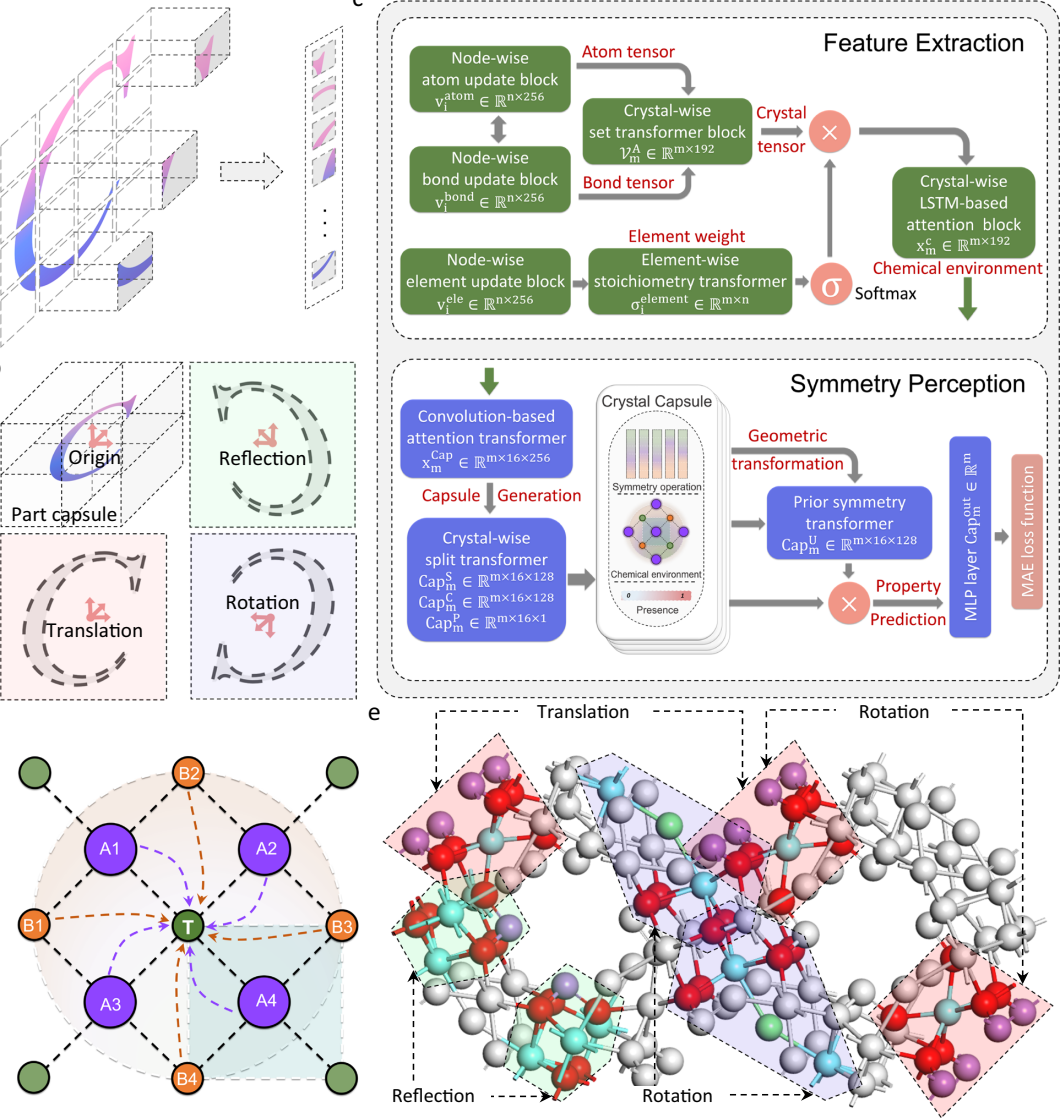
Development of the SEN (Symmetry-enhanced equivariance network) model to learn crystal symmetries and to predict material properties. **a** Schematic illustration of feature extraction based on the convolution operation. The left part represents the feature extraction through convolution kernels, and the right part represents the stacking computation scheme among features. **b** Schematic illustration of symmetry representation via the capsule operation. The symmetry correlation between objects can be directly identified via prior geometric transformations of part capsule module. **c** The SEN architecture comprised of the feature extraction, symmetry perception, and property prediction blocks, wherein $\sigma$ is the SoftMax activation function, $\bigotimes$ stands for the multiplication, LSTM is the long-short term memory model, MLP is the multilayer perceptron, and MAE is the mean absolute errors. d Schematic illustration of the chemical environment belonging to the central atom T, with the interaction domain and primitive cell denoted by the orange circle and blue rectangle respectively. **e** Equivariant structure patterns of the La_3_As_2_ClO_7_ crystal. The red, blue, and green regions present equivalent transformations including transition, rotation, and mirror.

Unfortunately, existing ML algorithms for crystal materials (including CGCNN, GATGNN, AMDNet and MegNet) based on advanced graph networks can hardly encode the rich invariance and equivariance, due to their unanimous foundation of conventional convolution neural networks^18–21^. While the inductive biases and weight sharing operations of the conventional convolution kernel preserve the translation symmetry^22–25^, such convolution and pooling operations inevitably forsake the rotation, inversion reflection, and mirror symmetries as illustrated in Fig. 1a. SchNet is the only ML model that perceived rotational invariance of crystals, yet such invariant network is not capable of describing the spatial relationship and correlation between equivalent clusters within a material^26,27^. A few architectures have been proposed to expand the Euclidean (E(n)) equivariance in molecular systems via implementing the learnable spherical harmonic kernel^28–30^ or the coordinate embedding method on the convolution filters^31^. Nevertheless, only the rotational equivariance has been considered in the molecular systems, and hence these models are insufficient to perceive the complicated crystal symmetries containing different types of spatial transformations and even finite combinations of them^30,32,33^. No significant improvement of prediction performance has been demonstrated by existing approaches via the partial recognition of material symmetries, and the underlying mechanism responsible for the impact of material symmetries on property prediction remains to be understood. (Detailed explanation of crystal symmetry is documented in Supplementary Note 1, literature summary of existing E(n) equivariant models are provided in Supplementary Note 2.)

Recently, the stacked capsule autoencoder (SCAE) model has received attention for precisely learning the full E(n) group equivariant properties of spatial patterns in graphics^34^. Diverse spatial symmetries can be successfully identified via deconstructing spatial features and incorporating sufficient priori geometric transformations to build the capsule representation of objects (Fig. 1b). The graphic model of crystals can be promoted into a capsule-based material representation by incorporating physical interpretations of its microscopic structures and atomic interactions. We speculate that the symmetry-based DL model combined with the capsule decoder can be developed to perceive equivalent sub-structures, which would enable the direct extraction of spatial symmetry features from original data without additional explicit descriptors and model training processes. Nevertheless, traditional capsule models are incapable of analyzing the complicated structure-property relationship of material systems. To realize the above promise, a fresh material simulation network needs to be designed for incorporating hierarchical structure and chemistry information, while appropriate transformer layers need to be established for predicting continuous material properties. These are challenging missions that have not yet been achieved in material science.

Herein, we developed a ML model named symmetry-enhanced equivariance network (SEN) to identify structure equivalences and thus to accurately predict properties of crystal materials. The designs of capsule representation with appropriate chemical environments are demonstrated to be crucial for symmetry recognition and material property predictions. The SEN overcomes the poor performance of convolution-based algorithms in the high-symmetry space groups, and achieves property predictions with high precision for materials in all space groups. The competitive performance of SEN along with its high interpretability and transferability unveils its potential for exploring the complicated and high-dimensional data in material science.

## Results

### Development of SEN to perceive crystal symmetries

Crystal symmetries can be described in ML as the appropriate set of equivariant transformations on structural patterns,

1 $$f\left(x\right)=f({Tx})$$

where $x$ represents the spatial patterns of crystals, $T$ is the spatial transformations related to crystal symmetry, and $f$ represents the non-linear discrete mapping to material properties. Taking advantage of the capsule mechanism for learning local equivariance and global invariance, we developed the SEN model to perceive crystal symmetries and to accurately predict material properties. The SEN adopts a complex DL architecture that encompasses the feature extraction, symmetry perception, and property prediction blocks as illustrated in Fig. 1c.

We first extracted the features of crystal materials based on the concept of chemical environment and the representation of graph models^18,21^. The chemical environment of a target atom is defined to represent the surrounding atoms and bonds within its cut-off radius (Fig. 1d). The atom type, atom connectivity, and bond lengths adjacent to each atom were extracted from the Materials Project (MP) database^35^. The combination of all atomic environments within the primitive cell was then encoded via the concatenation operator and the set2set transformer to represent the overall chemical environment of crystal material ${x}_{m}^{c}$^36^.

2 $${x}_{m}^{c}={{{{{{\mathcal{F}}}}}}}_{c}\left({x}_{m}^{{atom}},{x}_{m}^{{bond}}\right)$$

where ${x}_{m}^{{atom}}$ and ${x}_{m}^{{bond}}$ denote the information associated with atoms and bonds respectively, ${{{{{{\mathcal{F}}}}}}}_{c}$ is the transformer mapping to ${x}_{m}^{c}$. The chemical environment matrices of *N* atoms with 192 dimensions were trained by the feature extraction block consisting of multiple DL models.

In the second stage, we constructed a sufficient set of material capsules to perceive and inherit the crystal symmetry^34^, with each capsule composed of a symmetry operator, a convoluted material chemical environment, and a presence. The function of each material capsule can be roughly viewed as the critical feature extraction within chemical environment using a special capsule kernel that can be transformed with the symmetry operators, which satisfies

3 $${{{{{{{{\mathcal{T}}}}}}}_{c}{{{{{\mathcal{F}}}}}}}_{{cap}}\left({x}_{m}^{{Cap}}\right){{{{{\mathscr{=}}}}}}{{{{{\mathcal{F}}}}}}}_{{cap}}\left({{{{{{\mathcal{T}}}}}}}_{c}{x}_{m}^{{Cap}}\right)$$

where ${x}_{m}^{{Cap}}$ is a set of crystal capsules representing the material chemical environment, ${{{{{{\mathcal{T}}}}}}}_{c}$ is a symmetry operator that propagates the geometric transformations into the part capsules, ${F}_{{cap}}$ generates the updated crystal capsule incorporating the chemical environment and spatial information. The above equation implies that crystal symmetries have been identified and encoded before the projection to material property, and thus identical contributions from equivalent patterns would be expected for property predictions. The presence defined as the weight of each capsule is trained to effectively sample and screen the material capsules. Taking advantage of the capsule transformers, important multi-scale spatial patterns of crystals are propagated into the learning process for structure-property relationship as priori information (Fig. 1e), which ensures the equivariance for the symmorphic transformations (including translation, rotation, reflection, and mirror). Simultaneously, the equivalences between local clusters arising from the nonsymmorphic transformations (mixing transformations, including screw rotation and glide mirror) can be perceived by deconstructing material chemical environment to atomic clusters and propagating prior symmetry features.

Regarding the property prediction, we employed the variational statistical mechanisms to optimize the learning process and to obtain the probability distribution function (PDFs) of ${x}_{i}^{{atom}}$ and ${x}_{j}^{{bond}}$ ($i,j\in {R}_{N},{R}_{K}$). The SEN was trained by maximizing the likelihood function

4 $${{{{{\mathcal{L}}}}}}{{{{{\mathscr{=}}}}}}\mathop{\prod }\limits_{m}^{M}\mathop{\prod }\limits_{i}^{N}\mathop{\prod }\limits_{j}^{K}\left[P\left({y}_{m}{{{{{\rm{|}}}}}}{{{\varnothing }}}_{{cap}}\left({x}_{m}^{c}\right)\right)P\left({x}_{m}^{c}{{{{{\rm{|}}}}}}{x}_{m,i}^{{atom}},\, {x}_{m,j}^{{bond}}\right)\right]$$

where $i$ and $j$ denote the *i*th atom and the *j*^th^ bond in the *m*^th^ crystal, $N$ and $K$ are the number of atoms and the number of bonds in the *m*^th^ material. The conventional MAE loss is employed for our purpose of symmetry identification and single-point property prediction.

The performance of SEN was examined by the data set composed of 6027 crystal materials, which was obtained from the MP database and predicted by DFT calculations. The crystal data set covering all of the seven crystal systems was divided into the training, validation, and testing datasets at a ratio of 8:1:1. The atom embedding dimension and the number of capsules are 128 and 16, respectively. The SEN models were developed using the Python 3.7 and the TensorFlow framework^37^. Detailed algorithm architecture, data structure, loss function selection, and training process are documented in Supplementary Note 3 and Note 4.

### Material features encoded by chemical environment

The chemical environment of a material has been successfully applied to predict evolutionary configurations in ML-based molecular dynamics^6,11,20^. In particular, our material representation enables the learning of the contributions from both atoms and bonds, the distinction of strong chemical bonds from weak Van-der-Waals interactions, and the recognition of length dependent bond strength.

**Fig. 2:**
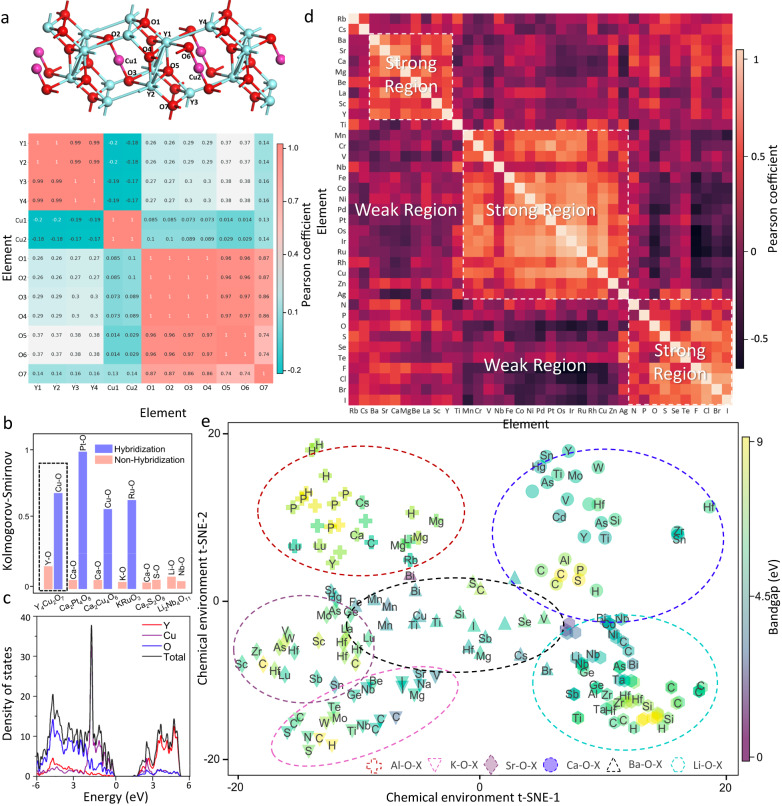
Feature extraction based on the material chemical environments. **a** Atom-based correlation analysis of chemical environments for Y_4_Cu_2_O_7_, wherein O, Cu, and Y atoms are respectively red, pink, and blue balls. **b** Atomic correlation between two elements of six materials learned by SEN (Symmetry-enhanced equivariance network), with the red and blue bars indicating the absence and occurrence of hybridization, respectively. **c** The PDOS (projected density of states) of Y_4_Cu_2_O_7_ crystal. **d** Element-based correlation analysis of chemical environments with compounds randomly selected from the MP (Material Projects) database. **e** Clustering analysis via calculating t-SNE (t-distributed stochastic neighbor embedding) on the chemical environments of A-O-X compounds. Six material groups are presented by different shapes (circles, equilateral triangles, inverted triangles, hexagons, diamonds, and plus signs), with the notation reflecting the X element. The color of the shape illustrates the bandgap value.

To demonstrate the effectiveness of our feature extraction model, we trained the SEN to predict the band gaps of crystal materials until the MAE is lower than 0.15 eV, and then analyzed the intermediate data of chemical environments produced by the feature extraction block. Specifically, we extracted the chemical environment matrices for individual atoms in the primitive cell of Y_4_Cu_2_O_7_. The Pearson coefficients between atom matrices were calculated to generate an atomic correlation heatmap as shown in Fig. 2a. According to the much larger coefficients between atoms within the same element group compared to those from different element groups, three element groups in Y_4_Cu_2_O_7_ can be clearly distinguished from the heatmap. For atoms within the same element group, the small differences in Pearson coefficients are consistent with their inequivalent local chemical environments.

The information of atomic interactions has been learned and encoded by our model. This is illustrated by calculating the 2-sample Kolmogorov-Smirnov (2-KS) of distinct atomic environments for six materials (Fig. 2b). The null hypothesis is that the two distributions are identical, and the alternative hypothesis is that they are not identical, which are justified by the low *p*-value. In the histogram plots, the y-axis is the normalized 2-KS value between the two atomic chemical environments belonging to different elements within the crystals. The projected density of states (PDOS) of Y_4_Cu_2_O_7_ is acquired by DFT simulation, wherein the larger overlap between the PDOS of Cu and O atoms near the band edges compared to that of the Y and O atoms (Fig. 2c) indicates the stronger interactions between the Cu and O atoms. By comparing the 2-KS and the PDOS plots for the six materials (Supplementary Fig. 3), we found that the orbital hybridization between two atoms around the fermi level occurs for all atom pairs with large 2-KS values (highlighted in blue), and vanishes for the atom pairs with small 2-KS values (highlighted in red). The results suggest that the hybridization phenomenon has been successfully detected by the SEN model, which is important for the predictions of electronic properties.

Generality and transferability of atomic chemical environments were investigated by building an elemental correlation heatmap for the entire 64 elements in the periodic table (the heatmap with 36 elements is shown in Fig. 2d for clarity, and the entire matrix is documented in Supplementary Fig. 4). Each correlation coefficient was computed for the chemical environments of two atoms randomly selected from the two crystal materials, while the materials were randomly selected from the MP database. The robustness of heatmap has been tested to justify the random selection process (Supplementary Fig. 5, 6). The heatmap exhibits similar trends to those in the periodic table, wherein the elements belonging to the same main group or with similar atomic numbers manifest comparable atomic environments. While most regions imply weak correlations, three blocks with strong elemental correlations can be clearly identified to present the groups of rare-earth metal, transition metal, and nonmetal.

The reliability of the overall material chemical environments was confirmed by probing the material correlations. Six material groups comprised of binary and ternary compounds in the form of A-O-X were selected, which contain 42 elements and 408 materials. The ‘A’ components include the Ca, Al, Ba, K, Sr, and Li elements, while the ‘X’ components span all main groups. By performing dimensionality reduction on the chemical environment matrices, we attained the two-dimensional t-distributed stochastic neighbor embedding (2D t-SNE) plot of the unsupervised cluster distribution patterns (Fig. 2e, only the materials with positive bandgaps are shown for clarity). The result shows that each material has a unique chemical environment, and the 408 materials form a wide dispersion in the critical feature space. Crystals with similar compositions exhibit analogous chemical environments, and thus the data are clearly divided into six cluster regions to present different material groups. The intersection areas between cluster regions can be explained by the similar chemical properties of the X elements.

### Interpretation and prediction based on equivariant representation

**Fig. 3:**
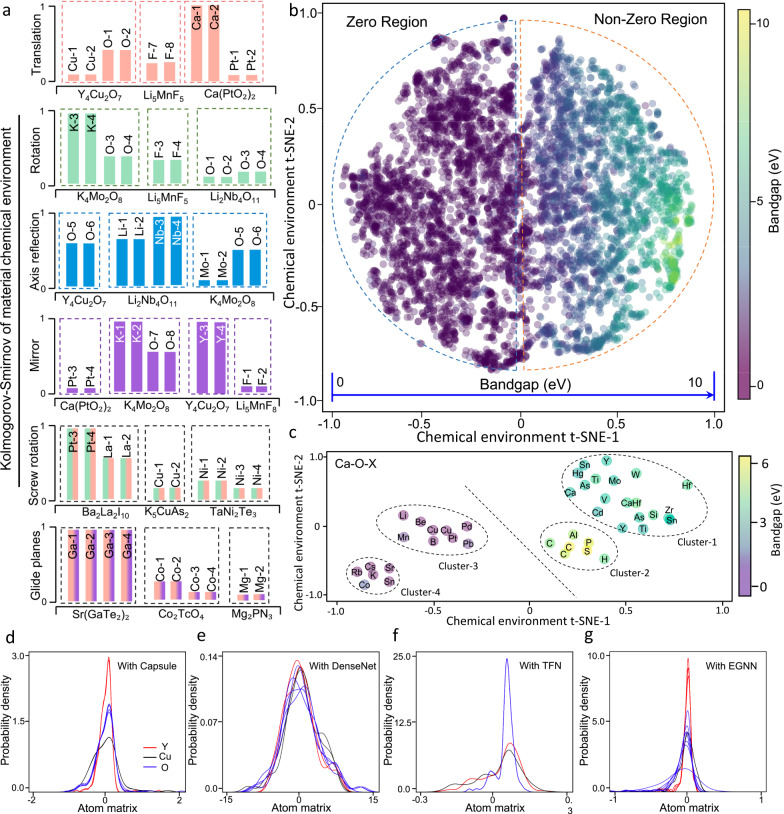
Interpretation and prediction based on equivariant representation. **a** Quantitative perception of crystal symmetries in representative crystal materials via the SEN (Symmetry-enhanced equivariance network). Each bar denotes the 2-KS (2 sample Komogorov-Smirnov) value of an atom referred to the same atom within the material, and the dashed boxes show the distinct materials. The six subgraphs present the perceived equivalent atoms arising from the six types of symmetric transformations (translation, rotation, inversion reflection, mirror, screw rotation, and glide mirror). **b** The 2D t-SNE (t-distributed stochastic neighbor embedding) plot of chemical environments with 6027 materials, with the color of circle denoting the bandgap value. **c** Cluster analysis on the chemical environments of Ca-O-X compounds. **d**–**g** PDFs of atomic chemical environments in Y_4_Cu_2_O_7_ obtained by SEN with capsule, DenseNet, TFN (Tensor field networks), and EGNN (E(n) equivariant graph neural networks) modules. The lines with the same color represent different atoms of the same element within the unit cell. The almost identical density distribution patterns among equivalent atoms and their distinctions from those of inequivalent atoms demonstrate the recognition of crystal symmetry **d**. Incomplete learning of crystal symmetry and atomic equivalence lead to chaotic distribution patterns **e**–**g**.

In order to accurately predict global material properties, we developed the capsule transformer for recognizing equivalent local patterns and learning crystal symmetries of space groups (Fig. 3). The effectiveness of our model was examined by comparing the chemical environments trained through the capsule transformer for all atoms within each material (Fig. 3a). From the PDF plots of chemical environments for all atoms in the primitive cell of Y_4_Cu_2_O_7_ (Fig. 3d), three classes of similar distribution patterns can be clearly identified to represent the three element groups. Quantitative 2-KS analyses were further conducted to verify the equivariance perception within each element group (Fig. 3a). The y-axis of the histogram plot presents the normalized 2-KS values referring to the same atom. Almost identical 2-KS values are observed for equivalent atoms caused by symmetric transformations, while the distinct 2-KS values appropriately reflect the inequivalent atoms in primitive cell. We extended the 2-KS calculations to material systems with full spatial transformations of different space groups. The equivariance among atoms originating from the translation, rotation, inversion reflection, mirror, screw rotation, and glide mirror operations are demonstrated by eleven materials (Y_4_Cu_2_O_7_, K_4_Mo_2_O_8_, Li_5_MnF_8_, etc.), consistent with the information in the MP database (crystal information and 2-KS values are documented in Supplementary Table 3).

To elucidate the role of capsule transformer in the symmetry perception block, we designed ablation studies via separately replacing the capsule block by two non-equivariant model (MLP and DenseNet)^38^ and three equivariant models (TFN^28^, SE(3)^30^, and EGNN^29^). For the cases with MLP and DenseNet (a state-of-the-art convolution neural network), the irregular PDF patterns exhibit no correlation with the intrinsic similarity between relevant atomic chemical environments (Fig. 3e). The poor performance of DenseNet probably arises from the irrational weighting on different types of material features in pooling layers. Regarding the cases with TFN (Fig. 3f) and SE(3) models, the vanishing diversity of atomic chemical environments prevents the recognition of equivalent atoms. For the case with EGNN (Fig. 3g), it is impossible to distinguish element groups and to identify equivalent atoms from the disorder PDF patterns. Detailed information is documented in Supplementary Note 6 and Note 8. The implementation of capsule transformer is thus demonstrated to be essential for learning crystal symmetries.

We then explored the mapping from chemical environments to material properties in our SEN model. Five materials were selected from the MP database, including Be_6_Ni_2_, Sr_4_Ge_2_S_8_, Li_2_V_2_F_12_, CsAsF_6_, and BaB_2_F_8_ with the bandgaps of 0 eV, 3.25 eV, 4.86 eV, 7.24 eV, and 10.12 eV, respectively. Strong correlation is observed between the bandgaps and the PDF patterns of material chemical environments, namely the PDF pattern gradually spreads with increasing bandgaps as illustrated in Supplementary Fig. 11. The overall projection from the material chemical environment to the bandgap for the entire dataset is presented through the 2D t-SNE plot in Fig. 3b. The 6027 materials are homogeneously distributed in the principal feature space, while the change of bandgaps is continuous and monotonic over the entire space (as seen from the color and node size distributions). The principal feature space can be divided into two regions encompassing materials with no bandgap (left) and with nonnegligible bandgap (right). Similar trends have been acquired in the 2D t-SNE plot for formation energy as shown in Supplementary Fig. 12. By contrast, the bandgap distributions in the 2D t-SNE plots obtained with the MLP and DenseNet models exhibit rough trends and large overlaps between materials with no bandgaps and those with large bandgaps (Supplementary Figs. 13, 14).

In order to verify that the feature-property relationship learned by our ML model conforms to the underlying physical principles, we generated the 2D t-SNE plot of chemical environments for the representative group of Ca-O-X materials (Fig. 3c). The dataset can be divided into four clusters in principal feature space. The 1st and 2nd clusters are comprised of semiconductors with medium and large bandgaps respectively, while both the 3rd and 4th clusters are comprised of metals with negligible bandgaps. By investigating a variety of material features (component, point group, spin polarization, etc. as documented in Supplementary Table 4), we found that the material bandgaps depend on complex features and can not be simply predicted by any critical factors^11,39^. Nevertheless, rational trends can be detected as follows to justify our model. While neither spin polarization nor orbital hybridization near the Fermi level is observed in semiconductor groups, each material in the metal groups possesses at least one of these two features. All the metals with orbital hybridization appear in the 3rd cluster, and the remaining metals with uniform m $\bar{3}$ m point group and significant spin splitting lie at the 4th cluster. As for the semiconductor materials, the two clusters can be roughly distinguished by the type of X atom as metal or non-metal. These trends have been confirmed by the complete distribution patterns of other compound groups (Supplementary Fig. 18).

**Fig. 4:**
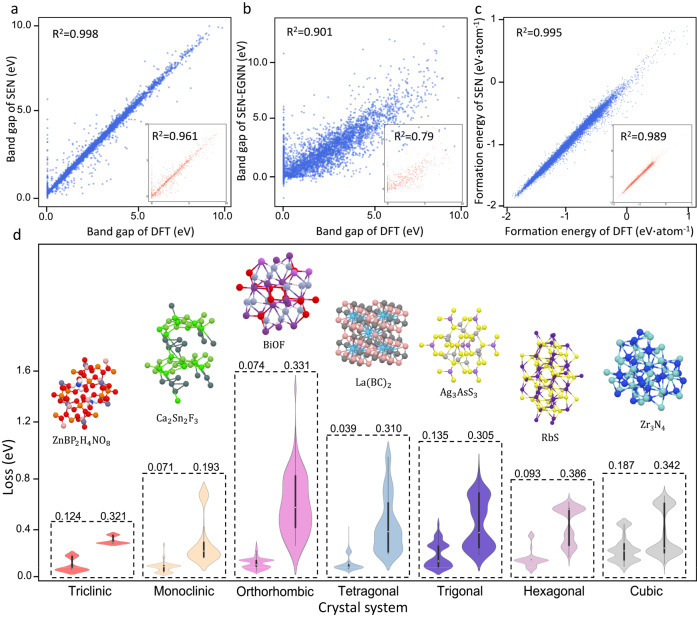
Prediction performances of material properties on crystals with various symmetries. **a**, **b** Prediction performances of bandgap by the SEN (Symmetry-enhanced equivariance network) and SEN-EGNN (SEN with E(n) equivariant graph neural networks) models, wherein R^2^ is the linear regression fitting coefficient. **c** Prediction performance of formation energy by the SEN. The blue and red scatters present the full and testing datasets respectively. **d** Comparison of prediction loss distributions via violin plots between the SEN and MegNet^21^ models for different crystal systems, wherein the left and right ones correspond to the SEN and MegNet models respectively. The width of the violin plot represents the distribution density of data, the black line-box plot within each violin illustrates the 25%, 50%, and 75% quartiles, and the thin line interval shows 95% confidence interval.

The bandgap prediction by the SEN model achieves remarkably low MAEs of 0.25 eV for testing set as illustrated in Fig. 4a, exhibiting a significant improvement (Fig. 4b) compared to the MAEs obtained by the models with the MLP, DenseNet, TFN, SE(3), and EGNN blocks (0.58 eV, 0.55 eV, 0.49 eV, 0.86 eV, and 0.76 eV for testing set). Detailed testing comparison of the five models is documented in Supplementary Fig. 19. Accurate prediction of formation energy is also accomplished with a low MAE of 0.0184 eV/atom for testing set as shown in Fig. 4c. We further conducted the unbiased test with the MatBench dataset^40^ (MAE reduction of SEN on bandgap/formation energy compared to CGCNN, MEGNet, and SchNet models: 39.1%/52.6%, 6.2%/36.1%, and 23.3%/26.1%) and the fair tests with identical datasets, which confirm that the prediction performance of the SEN model is improved compared to the other models (Supplementary Table 5-6). The significant impact of symmetry perception on property prediction was further revealed by inspecting the prediction quality for different crystal systems of various point group symmetries obtained by our SEN model and the MegNet^21^ model (Fig. 4d). From the box plots of error distributions, the prediction performance of SEN is much better than the MegNet in all crystal systems. The MegNet model performs worse in crystal systems with larger number of symmetric transformations (orthorhombic, tetragonal, trigonal, hexagonal, and cubic) than in low symmetry crystal systems (triclinic and monoclinic), while more significant improvements have been observed in high symmetry crystal systems by implementing the SEN model. Even with the SEN model, exceptionally large errors are observed in some materials, which are either magnetic systems with noticeable spin splitting or nonstoichiometric compounds with unusual bond orders (Supplementary Note 8).

## Discussion

From the ML perspective, the material properties mainly depend on the presence of important atomic clusters embedded in the crystal structure, the connectivity between clusters, and the structure-property relation determining the contribution of each cluster or cluster connection^18–21,41^. The recognition of equivalent clusters arising from crystal symmetry within a material is crucial for predicting electronic structure^29^. That is because the mapping from such clusters or relevant cluster interaction to the material property is appropriately restricted to be identical, which is consistent with physical theory. Meanwhile, the recognition of almost equivalent clusters across different materials enhances the effective data size and improves the prediction accuracy.

During the past few years, the property prediction models for crystal materials have been substantially improved by incorporating additional features such as global states (MEGNet^21^, GATGNN^19^) and atomic cluster (AMDNet^20^) based on the original crystal graphic model (CGCNN^18^). These methods cannot recognize crystal symmetries beyond translational invariance owing to the conventional CNN kernel. The performances of our SEN model on predicting bandgap and formation energy exceed those of the MEGNet, GATGNN, AMDNet, and CGCNN models (detailed comparisons can be found in Supplementary Note 9). This can be explained by the substantial reduction of effective feature dimensionality through the perception of the full crystal symmetries in the SEN model. Such feature purge process mitigates the overfitting problem and strengthens the mapping from material features to properties. The prediction accuracy of our SEN model on formation energy is also superior to that of the SchNet^27^ model with rotational invariance. The improvement can be attributed to both the recognition of additional crystal symmetries and the equivariance mapping. Even though both invariant and equivariant transformations can be employed to recognize the equivalence among clusters, only the equivariant transformations preserve the relative configurations among clusters by passing the symmetry operators. The spatial information preserved by equivariant transformation is essential for predicting the interaction strengths between clusters.

The successful merge of the crystal graphic model and capsule transformer can be understood with the above cluster scenario. The SCAE^34^ describes an image (object) as geometrical arrangements of parts, by discovering the important parts in an image and inferring their spatial relationships to the viewer (called the pose). The input is deconstructed into lots of part capsules, and each capsule contains a six-dimensional pose vector associated with the symmetry operation. The crystal material fits into the concept of object in the SCAE model, while the various types of equivariant atomic clusters embedded in the crystal structure correspond to the part capsules. The implementation of capsule transformer on the material chemical environment generates a sufficient set of part capsules representing critical local features, and enables the perception of complex equivariance by training the different pose vectors in different capsules. Such approach to chemical information processing enables the appropriate mapping of critical clusters and cluster interactions on material properties, which has not been achieved by prior studies.

In summary, we have developed the SEN model based on DL and capsule-based transformers to learn crystal symmetries and thus to accurately predict material properties. The MAEs obtained by the SEN model for predicting bandgap and formation energy are about 22.9% and 38.3% lower than those of prevalent ML models. Through quantitative analysis of intermediate training data, we found that such better performance can be attributed to both the establishment of material representation with joint structure-chemical patterns and the subsequent identification of pattern equivalence in different scales. The perception of cluster equivariances related to both the basic symmetric operations (translation, rotation, mirror, inversion reflection) and the mixing symmetric operations (screw rotation and glide mirror) are accomplished by the local feature extraction and the basic equivariant transformations. This work not only provides a general method to improve the prediction of arbitrary material properties, but also opens an opportunity to promote ML algorithms by implicitly encoding the relationship between material features based on the underlying physical mechanisms.

## Methods

### Framework of the SEN

In this work, we achieved accurate predictions of multiple material properties, and benefited from describing atomic clusters and cluster interactions via the SEN model. This is accomplished through the unified training of three modules including the feature extraction (FE), symmetry perception (SP), and property prediction (PP) blocks.

The FE block perceives the input data of atoms and bonds to construct the chemical environment ${x}_{m}^{c}$ of target material following the standard procedure developed by Xie and Grossman^18^. The material datasets composed of the stoichiometry, crystal structure, atom information, and bond information are constructed by a high throughput screening process (Detailed data structure is documented in Supplementary Note 3). The crystal information files (CIFs) of the MP database are filtered by leveraging the Pymatgen^35^ interface of material genetic engineering as shown in Box 1.

The input data includes the information of N atoms and M bonds in the primitive cell of target material. The ${{{{{{\mathcal{F}}}}}}}_{c}$ transformer models possess three different attention-based encoders that produce atomic tensors and bond tensors with (N×256) dimensions. The ${SET}$ is a set transformer based on concatenation operation to build atomic chemical environment with (N×192) dimensions incorporating atom and bond information. The ${{Att}}_{{Sto}}$ is a stoichiometry transformer in terms of an attention-based weight reconstruction model to describe the element correlations. The ${{Att}}_{{LSTM}}$ is a LSTM-based attention model to encode multi-range correlations between atoms.

With the material datasets as the only input of the SEN model, we simultaneously calculate the atomic chemical environment vectors ${{{{{{\mathscr{V}}}}}}}_{m}^{A}$ and the elemental weight vectors ${V}_{m}^{E}$ based on the structure data (${x}_{m}^{{atom}}$ and ${x}_{m}^{{bond}}$) and the stoichiometric data (${x}_{m}^{{atom}}$) respectively. The atomic chemical environment matrices ${{{{{{\mathscr{V}}}}}}}_{m}^{A}$ represent the complex correlations between each target atom of the primitive cell and its surrounding atoms/bonds within the cut-off radius, which is calculated with a concatenation operator and a set2set transformer^36^.

5 $${{{{{{\mathcal{V}}}}}}}_{m}^{A}={SET}\left\{\mathop{\sum}\limits_{N}{\left[{{{{{{\mathcal{F}}}}}}}_{c}^{a}\left({x}_{i}^{{atom}}\right)\bigoplus {{{{{{\mathcal{F}}}}}}}_{c}^{b}\left({x}_{i}^{{bond}}\right)\left.\bigg)\right.\right]}_{i\in N}\right\}$$

where ${{{{{{\mathcal{F}}}}}}}_{c}^{a}$, ${{{{{{\mathcal{F}}}}}}}_{c}^{b}$ are the atomic transform mapping and bond transform mapping in the chemical environment mapping ${{{{{{\mathscr{F}}}}}}}_{c}$, $\bigoplus$ is the concatenation operator.

The elemental weight vectors ${V}_{m}^{E}$ represent the reconstruction of elemental influence towards target property via the stoichiometry transformer^25^.

6 $${V}_{m}^{E}={softmax}\, \left\{\mathop{\sum}\limits_{N}{\left[{{Att}}_{{Sto}}\left({x}_{i}^{{atom}}\odot {x}_{j,j\ne i}^{{atom}}\right){{{{{{\mathcal{F}}}}}}}_{c}^{e}\left({x}_{i}^{{atom}},{x}_{j,j\ne i}^{{atom}}\right)\right]}_{i,j\in N}\right\}$$

where ${{{{{{\mathscr{F}}}}}}}_{c}^{e}$ is the elemental transform mapping in the chemical environment mapping ${{{{{{\mathscr{F}}}}}}}_{c}$, $\odot$ is the node-wise matrix operation between the target atom and its surrounding atoms, and ${softmax}$ is the probability activation function^42,43^. ${{Att}}_{{Sto}}$ is the stoichiometry transformer redesigned as an attention encoder based on graph model, which performs element weight calculation by receiving the atomic information within the cut-off region.

After activation with the multi-layer perceptron, the elemental weight vectors are transformed into the probability vectors of the corresponding atoms. Updating all atomic level correlation by an element-wise operation between the atomic chemical environment vectors and the elemental weight vectors, we obtain the chemical environment matrix ${x}_{m}^{c}$ of the material via the LSTM-attention layers.

7 $${x}_{m}^{c}={{Att}}_{{LSTM}}\left({{{{{{\mathcal{V}}}}}}}_{m}^{A}\otimes {V}_{m}^{E}\right)$$

where ${{Att}}_{{LSTM}}$ is the redesigned attention block incorporating the LSTM model, and $\otimes$ is the node-wise multiplier.

The material chemical environment is then transformed into the material capsules composed of a symmetry operator, a convoluted material chemical environment, and a presence value via the decoder of SP block (Supplementary Fig. 2). The diverse symmetry patterns are propagated to crystal capsules by implementing the symmetric operations on the material chemical environment matrices. The primary capsule layer of symmetry perception consists of a convolution-based attention (${{Att}}_{{Conv}}$) layer and a geometric transformation (Geo-Trans) layer as shown in Box 2. This convolution-based primary encoder is a series of three convolution layers with 256 filters, (3$\times$3) kernel sizes, and (2$\times$2) strides.

Herein, the ${{Att}}_{{Conv}}$ is a convolution-based attention transformer that outputs 16 part capsules with (16$\times$128) dimensions to deconstruct the diverse spatial patterns of material. The ${F}_{f}^{{split}}$ is a split transformer that outputs a capsule set (*N* = 16) of the material via perceiving chemical environment from the part capsules. The ${{{{{{\mathcal{T}}}}}}}_{c}$ is a symmetry operator that propagates the geometric transformations into the part capsules, mainly including scaling, translation, rotation, inversion reflection, and mirroring reflection transformations. The ${F}_{{cap}}$ is a MLP model to perform property prediction. The detailed algorithm architecture of SP block is provided in Supplementary Note 4.

The ${{Att}}_{{Conv}}$ layer first identifies multiscale spatial patterns in the crystal structure. The contribution weights of patterns are then updated by the attention mechanism modified with convolution operation.

8 $${{Cap}}_{m}^{S},\, {{Cap}}_{m}^{C},\, {{Cap}}_{m}^{P}={{F}_{f}^{{split}}}\left[{Att}_{{Conv}}\left({x}_{m}^{c}\right)\right]$$

where ${{Cap}}_{m}^{S},{{Cap}}_{m}^{C},{{Cap}}_{m}^{P}$ are the symmetry operators, chemical environment, and presence value respectively in each material capsule, ${{Att}}_{{Conv}}$ is the redesigned attention block incorporating the convolution operation, and ${F}_{f}^{{split}}$ is the split transformer. In contrast to the straight splitting employed in standard SCAE, an attention-based splitting layer is designed in our SEN model to rationally separate the spatial and chemical information, which is a self-attention layer with 192 neural nodes.

The Geo-Trans layer performs spatial transformation operations (including translation, rotation, reflection, scale, and shear) on all detected spatial patterns, and propagates the equivariances between transformed patterns to the capsule operator in SP block in the form of prior information.

9 $${{Cap}}_{m}^{U}={{{{{{\mathscr{T}}}}}}}_{c}({{Cap}}_{m}^{S},\, {{Cap}}_{m}^{C})$$

where ${{Cap}}_{m}^{U}$ is the updated material capsule incorporating the diverse symmetrical transformation patterns, and ${{{{{{\mathscr{T}}}}}}}_{c}$ is the symmetry operator. Geometric operations associated with the E(n) transformation are performed on spatial features for encoding the symmetry operator to crystal capsule, which is similar to the operation in standard SCAE. Based on the above extraction of feature correlations, the equivalent clusters arising from all crystal symmetries can be accurately identified from the updated cluster capsule representation, because the relevant vector parts of chemical environments and spatial features in the updated capsules associated with equivalent clusters should be identical. To compliant with the perceived correlation between clusters, the presence probabilities of all capsules were updated to appropriately represent the contribution weights of clusters. Finally, the mapping from key cluster contributions to property was established through the simple MLP network (${F}_{{cap}}$). Even though the MLP by itself can not perceive interactions between capsules, our model indeed account for the contribution from cluster interactions to predicted target property, because the capsule correlations obtained by the attention mechanism have been embedded in the updated capsule representation. The correlations between material capsules are then reorganized by passing the weighted information of the presence vector to the material capsules via an element-wise operator. The outputs of the capsule operations eventually serve as the inputs for the property prediction block to enable the accurate learning of the feature-property relationship. Detailed algorithm design and training process are provided in Supplementary Note 4.

Consistent with the data source in previous machine learning studies on crystal materials^18–21^, the datasets for predicting bandgap and formation energy in this work are sampled from the Materials Project (MP) database. The datasets for bandgap and formation energy contain 6027 and 30,000 materials, respectively. Both datasets are composed of 64 elements, which cover the entire periodic table except for the noble gases group, lanthanides, actinides, and radioactive elements.

Box 1 **Algorithm 1** Feature extraction block **Input:** atom ${x}_{m}^{{atom}}=({a}_{1},\ldots,{a}_{n})\in {M}^{n}$, ${x}_{m}^{{bond}}=({b}_{1},\ldots,{b}_{k})\in {M}^{k}$

**Trainable transformer:** $${{{{{{\mathscr{F}}}}}}}_{c}=({{{{{\mathscr{F}}}}}}_{c}^{a},{{{{{{\mathscr{F}}}}}}}_{c}^{b},{{{{{{\mathscr{F}}}}}}}_{c}^{e})$$, $${SET}(\cdot )$$, $${{Att}}_{{Sto}}(\cdot )$$, $${{Att}}_{{LSTM}}(\cdot )$$

**Output:** $${x}_{m}^{c}=({x}_{1}^{c},\ldots,{x}_{m}^{c})\in M$$

${{{{{{\boldsymbol{v}}}}}}}_{{{{{{\boldsymbol{i}}}}}}}^{{{{{{\boldsymbol{atom}}}}}}}\leftarrow {{{{{{\mathscr{F}}}}}}}_{c}^{a}({x}_{i}^{{atom}})$                       for all atoms $i$ in input crystal

${{{{{{\boldsymbol{v}}}}}}}_{{{{{{\boldsymbol{j}}}}}}}^{{{{{{\boldsymbol{bond}}}}}}}\leftarrow {{{{{{\mathscr{F}}}}}}}_{c}^{b}({x}_{j}^{{bond}})$                       for all bonds $j$ in input crystal

$${{{{{{\mathscr{V}}}}}}}_{{{{{{\boldsymbol{m}}}}}}}^{{{{{{\boldsymbol{A}}}}}}}\leftarrow {SET}(({v}_{1}^{{atom}},\ldots,{v}_{i}^{{atom}})\oplus ({v}_{1}^{{bond}},\ldots,{v}_{j}^{{bond}}))$$                        $$\forall i,j$$

$${{{{{{\boldsymbol{v}}}}}}}_{{{{{{\boldsymbol{i}}}}}}}^{{{{{{\boldsymbol{element}}}}}}}\leftarrow {{{{{{\mathscr{F}}}}}}}_{c}^{e}({x}_{i}^{{atom}},{x}_{k,k\ne i}^{{atom}})$$                               $$\forall i,k$$

$${{{{{{\boldsymbol{\sigma }}}}}}}_{{{{{{\boldsymbol{i}}}}}}}^{{{{{{\boldsymbol{element}}}}}}}\leftarrow {{Att}}_{{Sto}}\left({x}_{i}^{{atom}}\odot {x}_{k,k\ne i}^{{atom}}\right)$$                             $$\forall i,k$$

$${{{{{{\boldsymbol{V}}}}}}}_{{{{{{\boldsymbol{m}}}}}}}^{{{{{{\boldsymbol{E}}}}}}}\leftarrow {softmax}({v}_{i}^{{element}}\bullet {\sigma }_{i}^{{element}})$$                             $$\forall i$$

$${{{{{{{\boldsymbol{x}}}}}}}_{{{{{{\boldsymbol{0}}}}}}}\leftarrow {{{{{\mathscr{V}}}}}}}_{m}^{A}\otimes {V}_{m}^{E}$$                                     $$\forall m$$

$${{{{{{\boldsymbol{x}}}}}}}_{{{{{{\boldsymbol{m}}}}}}{{{{{\boldsymbol{,}}}}}}{{{{{\boldsymbol{t}}}}}}{{{{{\boldsymbol{=}}}}}}{{{{{\boldsymbol{0}}}}}}}^{{{{{{\boldsymbol{c}}}}}}}{\leftarrow x}_{0}$$

**For** t iterations **do**

 $${{{{{{{\boldsymbol{x}}}}}}}_{{{{{{\boldsymbol{m}}}}}}{{{{{\boldsymbol{,}}}}}}{{{{{\boldsymbol{t}}}}}}}^{{{{{{\boldsymbol{c}}}}}}}\leftarrow {Att}}_{{LSTM}}\left({x}_{m,t}^{c}\right)$$

**End for**

$${{{{{{\boldsymbol{x}}}}}}}_{{{{{{\boldsymbol{m}}}}}}}^{{{{{{\boldsymbol{c}}}}}}}\leftarrow {x}_{m,T}^{c}$$                                        $$\forall m$$

Return $${{{{{{\boldsymbol{x}}}}}}}_{{{{{{\boldsymbol{m}}}}}}}^{{{{{{\boldsymbol{c}}}}}}}{{{{{\boldsymbol{=}}}}}}{{{{{\boldsymbol{(}}}}}}{x}_{1}^{c},\ldots,{x}_{m}^{c}{{{{{\boldsymbol{)}}}}}}$$

Box 2 **Algorithm 2** Symmetry perception block **Input:** chemical environment ${x}_{m}^{c}{{{{{\boldsymbol{=}}}}}}{{{{{\boldsymbol{(}}}}}}{x}_{{m}_{1}}^{c}{{{{{\boldsymbol{,}}}}}}\ldots {x}_{{m}_{n}}^{c}{{{{{\boldsymbol{)}}}}}}$

**Trainable transformer:** $${{Att}}_{{Conv}}(\cdot )$$, $${F}_{f}^{{split}}(\cdot )$$, $${{{{{{\mathscr{T}}}}}}}_{c}(\cdot )$$, $${F}_{{cap}}(\cdot )$$

**Output:** $${y}_{m}=({y}_{1},\ldots,{y}_{m})\in M$$

$${{{{{{\boldsymbol{x}}}}}}}_{{{{{{\boldsymbol{m}}}}}}}^{{{{{{\boldsymbol{Cap}}}}}}}\leftarrow {{Att}}_{{Conv}}({x}_{m}^{c})$$                     $$\forall m$$

$${{{{{{\boldsymbol{Cap}}}}}}}_{{{{{{\boldsymbol{m}}}}}}}^{{{{{{\boldsymbol{S}}}}}}}{{{{{\boldsymbol{,}}}}}}{{{{{{\boldsymbol{Cap}}}}}}}_{{{{{{\boldsymbol{m}}}}}}}^{{{{{{\boldsymbol{C}}}}}}}{{{{{\boldsymbol{,}}}}}}{{{{{{\boldsymbol{Cap}}}}}}}_{{{{{{\boldsymbol{m}}}}}}}^{{{{{{\boldsymbol{P}}}}}}}\leftarrow {F}_{f}^{{split}}({x}_{m}^{{Cap}})$$

$${{{{{{\boldsymbol{Cap}}}}}}}_{{{{{{\boldsymbol{m}}}}}}}^{{{{{{\boldsymbol{U}}}}}}}\leftarrow {{{{{{\mathscr{T}}}}}}}_{c}({{Cap}}_{m}^{S},{{Cap}}_{m}^{C})$$

$${{{{{{\boldsymbol{\sigma }}}}}}}_{{{{{{\boldsymbol{m}}}}}}}^{{{{{{\boldsymbol{cap}}}}}}}\leftarrow {{Cap}}_{m}^{U}\otimes {{Cap}}_{m}^{P}$$

$${{{{{{\boldsymbol{Cap}}}}}}}_{{{{{{\boldsymbol{m}}}}}}}^{{{{{{\boldsymbol{out}}}}}}}\leftarrow {F}_{{cap}}({\sigma }_{m}^{{cap}})$$

Return $${{{{{{\boldsymbol{Cap}}}}}}}_{{{{{{\boldsymbol{m}}}}}}}^{{{{{{\boldsymbol{out}}}}}}}=({{Cap}}_{1}^{{out}},\ldots,{{Cap}}_{m}^{{out}})$$

### Supplementary information

Supplementary Information

Peer Review File

### Source data

Source Data

## Data Availability

The crystal symmetry and chemical environment data generated in this study have been deposited in the Zenodo database under accession code 10.5281/zenodo.8142678. The processed crystal symmetry and chemical environment data are available at Zenodo. The crystal symmetry and chemical environment data generated in this study are provided in the Supplementary Information/Source Data file. The crystal symmetry and chemical environment data used in this study are available in the Zenodo database under the accession code 10.5281/zenodo.8142678. Source data are provided in this paper^44^. Source data are provided in this paper.

## Code availability

The source code and associated trained models of the SEN are publicly available from Zenodo repository^44^ and GitHub at https://github.com/MatMLlab/ SEN_model ^45^.
