# Supplementary material for: Material symmetry recognition and property prediction accomplished by crystal capsule representation
Source: Nat Commun. 2023 Aug 25;14:5198. doi: 10.1038/s41467-023-40756-2 (PMC10457372; doi:10.1038/s41467-023-40756-2)
Supplement: Supplementary file 1 — Supplementary Information [file 41467_2023_40756_MOESM1_ESM.pdf]

Supplementary Information

Author Information

Affiliations

1.School of Physics, Sun Yat-Sen University, Guangzhou, 510275 China

2.Guangdong Provincial Key Laboratory of Magnetoelectric Physics and Devices, School of Physics, Sun Yat-sen University, Guangzhou 510275, China

3.Center for Neutron Science and Technology, School of Physics, Sun Yat-sen University, Guangzhou 510275, China

Chao Liang[1], Yilimiranmu Rouzhahong[1], Caiyuan Ye[1], Chong Li[1] , Biao Wang[1]*, and Huashan Li[1,2,3]*

Supplementary Information

Supplementary Note 1: The equivariance in physical systems

For comprehensively elucidating the relation between crystal symmetry and equivariant models, we discussed from the following three aspects: (1) the crystal symmetry and the Euclidean (E(n)) group; (2) the equivariance in physical systems; (3) the equivariance proof of the SEN model.

(1) The crystal symmetry and the Euclidean group

The crystal symmetry describes the intrinsic spatial features of the material microscopic structure with ordered arrangement of atoms, ions or molecules[1,2]. The concept of crystal symmetry has been widely implemented to reflect the equivalence among the embedded atomic clusters as well as the global similarity between different materials. The symmetry properties are concretized via the space group and point group. The point group is defined as a group of geometric symmetries with at least one fixed point, while the space group is defined as the set of all coordinate transformations that map the equilibrium positions of an infinite crystalline solid into itself[24]. The space groups are sub-groups of the Euclidean group.

Based on the relevant set of point groups, all crystal materials are classified into seven crystal systems including the triclinic, monoclinic, orthorhombic, tetragonal, trigonal, hexagonal, and cubic systems. With the consideration of additional translation operation, the seven crystal systems can be further divided into 230 space groups, which are categorized into the symmorphic and nonsymmorphic transformations. The former is composed of basic transformations including translation, rotation, reflection, and mirror. The nonsymmorphic class is composed of mixing transformations (sequential combination of basic transformations) including screw rotation and glide mirror. (Supplementary Table 1).

Supplementary Table 1. The relation between three-dimensional crystal families.

Crystal system	Required symmetries of the point group	Point groups	Space groups
Triclinic	None	2	2
Monoclinic	1 twofold axis of rotation or 1 mirror plane	3	13
Orthorhombic	3 twofold axes of rotation or 1 twofold axis of rotation and 2 mirror planes	3	59
Tetragonal	1 fourfold axis of rotation	7	69
Trigonal	1 threefold axis of rotation	5	7<br>18
Hexagonal	1 sixfold axis of rotation 4	7	27
Cubic	4 threefold axes of rotation	5	36
7	Total	32	230

A Euclidean group is the group of (Euclidean) isometries in a Euclidean space, that

is, the transformations of that space that preserve the Euclidean distance between any two points[3]. The E(n) group comprises all translations, rotations, reflections, and arbitrary finite combinations of them. The transformations of the E(n) group encompass all the transformations related to crystal symmetries. The crystal symmetry groups inherent the properties of the E(n) groups, such as dimensionality, topology, and affine transformation[4]. However, the crystal symmetry group is not identical to the Euclidean group, because it requires not only the preservation of E(n) distance but also the preservation of atom and bond type distributions. In this sense, the crystal symmetries contain more information than those associated with the spatial symmetries in the E(n) group, and thus their recognition and encodement demand much more complicated machine learning model than those targeting at the E(n) group.

A lot of convolution networks have been proposed to recognize the E(n) group by encoding the rotation and reflection equivariances among different objects[5-7]. To comprehensively perceive the symmetry properties of a single material, a collection of equivariances regarding reflection, inversion, improper rotation, screw rotation, and glide translation inside the crystal itself should be encoded as prior information[4].

(2) The equivariance in physical systems

The concepts of the invariant and equivariant phenomena in physical systems require clarification to avoid confusion to audiences. As a relatively rough but practical approximation for machine learning model, the material properties mainly depend on the presence of important atomic clusters embedded in the crystal structure, the connectivity between clusters, and the structure-property relation determining the contribution of each cluster or cluster connection. The recognition of equivalent clusters arising from crystal symmetry within a material is crucial for predicting electronic structure. That is because the mapping from such clusters or relevant cluster interaction to material property is appropriately restricted to be identical, which is consistent with the physical theory. Meanwhile, the recognition of almost equivalent clusters across different materials enhances the effective data size and improves the prediction accuracy. Without the equivalent cluster perception, the deep neural network is likely to suffer from the overfitting problem and converges to unrealistic solutions.

In our model, the cluster features are extracted by constructing atomic chemical environment for each atom, which incorporates the atom type, atom coordinates, bond length, bond index, material stoichiometry, and spatial information within the cutoff radius. Based on the above analysis, the overall structure-property mapping should be an invariant predictor regarding the crystal symmetric operations to reflect the identical contributions from equivalent components. However, the intermediate transformations should be equivariant rather than invariant. Even though both invariant and equivariant transformation can be employed to recognize the equivalence among clusters, the invariant transformations abandon any difference between the clusters, while the equivariant transformations preserve the relative configurations among clusters by passing the symmetry operators. The spatial information preserved by equivariant transformation is essential for predicting the interaction strengths between clusters.

(3) The equivariance proof of the SEN model

In this part we prove that our model is transformation equivariant on x for any transformation group $G \in E(n)$, for which $G = \{t, M\}$ mainly includes translation equivariant on x for any translation vector $t \in \mathbb{R}^n$ and rotation, mirror, and reflection equivariant on x for any orthogonal matrix $M \in \mathbb{R}^{n\times n}$. Formally, the model should satisfy the equivariant process:

$$MX_p^{l+1} + t, X_q^{l+1} = SEN(MX_p^l + t, X_q^l) \quad \text{(S1)}$$

wherein X_p^l and X_q^l are respectively atom positions and other elements of embedding vectors for all atom at layer l, $X_p^l = \{x_{p,i,j}^l\}_{i=1,2,3;j=1,\dots,N}$, and $X_q^l = \{x_{q,j}^l\}_{i=1,\dots,N}$, N is the atom number in crystal. The following discussion shows how the crystal symmetry propagates through our model, mainly including translation, rotation, mirror, and reflection. Firstly, the construction process of material chemical environment $x_m^c = \mathcal{F}_c(x_{p,m}^{atom}, x_{q,m}^{bond})$ via the attention-based encoder $\mathcal{F}_c$ propagates the complete set of spatial and chemical information including crystal symmetry[6,8].

The material chemical environment is then propagated through the capsule transformer to generate material capsules $Cap_m(X_p, X_q) = \phi_{cap}(x_m^c)$. The capsule transformation on geometric information has been demonstrated to possess E(n) equivariant[9].

$$F_{cap}\big(MCap_m(X_p) + t\big) = M'F_{cap}\big(Cap_m(X_p)\big) + t' \quad \text{(S2)}$$

where F_{cap} is the capsule mapping functions in symmetry perception block. In addition, the X_q^l comprised of atom type, bond connectivity, and bond lengths is invariant to $E(n)$ transformations since none of these quantities changes with ridge transformation. Therefore, we have

$$F_{cap}\big(MCap_m(X_p, X_q) + t\big) = M'F_{cap}\big(Cap_m(X_p, X_q)\big) + t' \quad \text{(S3)}$$

where M, M', t, and t' belong to the same transformation group $G \in E(n)$. Therefore, the SEN model can intelligently propagate the equivalent spatial patterns and maintain the equivariance of network.

We have proved that our SEN model is equivariant to the symmorphic transformations (basic transformations) since the spatial operations are directly encoded as prior features via the capsule mechanism. While the SEN model is not equivariant to nonsymmorphic transformation, the equivalences between local clusters arising from the nonsymmorphic transformations can still be perceived by deconstructing material chemical environment to atomic clusters and propagating prior symmetry features.

Supplementary Note 2: The introduction for existing E(n) equivariance models

Deep convolutional neural networks (CNNs) adopt convolutional weight sharing strategy and thus possess translation symmetry. Conventional CNNs have been strictly proved to be equivariant to translation, but not to other transformations such as rotation, reflection, etc.[5] To overcome this challenge, a lot of advanced CNNs have been

proposed to expand E(n) equivariance[6,7,10,11-16].

Group equivariant convolutional neural networks (Cohen & Welling, 2016) employed G-convolutions with a substantially higher degree of weight sharing than regular convolution layers to expand the p4 and p4m transformations[5]. Rotation equivariant vector field networks (RotEqNet et al., 2017) incorporated a modified convolution operator relying on the vector field representation to encode rotation equivariance, invariance and covariance[13]. Harmonic networks (H-Nets, Brostow et al., 2017) designed the patch-wise 360◦-rotational equivariance into deep image representations, by constraining the filters to the family of circular harmonics[14]. SE(3)-transformer (Fuchs et al., 2020) recompiled the self-attention to operate on graphs with varying number of points, while guaranteeing SE(3)-equivariance for robustness[6]. LieConv (Finzi et al., 2021) proposed a convolutional layer that is SE(3) equivariant to a given Lie group by defining exp and log maps[7]. These works focus on the rotation equivariance or specific group equivariance of convolution model, and is insufficient to perceiving the complex crystal symmetry with a combination of translation, rotation, reflection, mirror, screw rotation, and glide mirror operations. Besides, these approaches are designed for manipulating the continuous images, and thus can not be directly applied to the graphic material representation containing both spatial and chemical information.

Only a few studies have been conducted to expand E(n) equivariance in material systems. Tensor field network (TFN) (Thomas et al., 2018) successfully propagates the node embeddings and applies filters built from learnable spherical harmonics kernel which preserves SE(3) equivariance but is expensive to compute[15]. Tests on the QM9 molecular database were conducted to recover molecular structures after randomly removing an atom from the original structures. However, the pairwise atomic distances rather than the atom coordination are used as input, which is naturally invariant in spherical harmonics representation. Both the material symmetry perception and accurate property prediction have not been demonstrated in this study. E(n)-equivariant graph neural networks (EGNNs) (Satorras et al., 2022) present a novel architecture to combine graph neural networks and coordinate embeddings, which achieves translation, rotation, and reflection equivariant (E(n)) as well as permutation equivariant with respect to an input set of points[16]. This work also carried out tests on QM9 molecular database and achieved good performance. Nevertheless, as stated in their paper, coordinate embeddings and updating are not used in the molecular property prediction process, so it is impossible to verify whether the E(3) equivariance is achieved in the process.

In all, the advanced convolution network can be equivariant to the E(n) group, and previous studies in this area indeed offer us valuable perspectives for developing our SEN model for crystal materials. We also carefully learned the work of Kondor and Trivedi, which strictly demonstrates the necessity of convolutional architecture for equivariant learning under the conditions of compact group and translation group[17]. Accordingly, the translation equivariance learned by our model should be ascribed to

the convolution modules within the network, while the other types of equivariance is learned through the other modules within the capsule transformer.

Even though the advanced deep learning networks have the potential to perceive all symmetry in the E(n) group, most of them encode only part of the equivariance that associated with the E(n) group. In order to recognize the equivariant patterns associated with complex material symmetry, not only the equivariance of entire E(n) requires to be encode, but also the chemical information needs to be appropriately embedded in the equivariant criterion. The chemical information of our work can be divided into two parts: (1) the explicit features of microscopic structure including the atom type, atom coordinates, bond length, bond index, and material stoichiometry; (2) the constraints on advanced physical features, such as distributions of atomic interaction strengths, energy bands, and density of states.

In principle any models that cover the entire E(n) group can deal with the symmetry transformations in space groups. Recently, a few studies based on the spherical harmonics kernel[6,15] or coordinate embedding scheme[16] unveil the possible avenues to encode material equivariance using the molecular database. Unfortunately, most of the existing methods achieve equivariances regarding only one or two types of transformations (translation and rotation) within the E(3) group. To the best of our knowledge, none of them is capable of learning the equivariances of the full E(3) group, and none of them can perceive the symmetries related to the mixing transformations (e.g. the operation of translation followed by rotation). Since the symmetry group of a crystal material typically contain a large set of spatial transformations including translation, rotation, mirror, inverse reflection, and various mixing transformations (such as screw rotation and glide reflection), existing methods are impossible to comprehensively perceive the crystal symmetry. And how the equivariance perception affects the material property prediction remains to be understood. For filling this knowledge gap, the above challenges are addressed by merging the advanced machine learning algorithm and the physical insight on structure-property relationship. Our key idea is to develop a ML model that reflects the underlying physical principle: the material properties mainly depend on the important atomic clusters embedded in the microscopic structure, the connectivity between clusters, and the structure-property relation determining the contribution of each cluster or cluster connection. Therefore, we develop the SEN model to identify structure equivalences and to accurately predict properties of crystal materials (with much more complex symmetry than molecules).

Similar to the advanced graphic models for material property prediction[18-21], the input chemical information is processed to rationally establish atomic chemical environments and material chemical environments. Regarding the advanced physical features, they are formidable to be predicted by ML models yet crucially affect the material properties. Since most of the advanced physical features are approximately determined by the characteristics of important clusters and the interactions between these clusters, despite the constrains on advanced physical features cannot be explicitly learned, such constrains can be reflected to the constrains on the mapping from clusters

to material properties. To this end, we designed the symmetry perception block based on capsule mechanism to deconstruct the crystal representation into important clusters, to propagate the prior features of symmetry transformations, and to preserve the spatial relationships between clusters. Such new approach of chemical information processing enables the appropriate mapping of critical clusters and cluster interactions on material properties, which has not been achieved by prior studies.

The perception of crystal symmetry may improve the material property prediction by identifying equivalent and inequivalent clusters, which substantially reduces the feature space and enables more accurate mapping from cluster to property. Such speculation is realized through developing the SEN model based on chemical environment representation and capsule mechanism. The implementation of capsule transformer on the material chemical environment generates a sufficient amount of part capsules representing critical clusters. The perception of cluster equivariances related to both the basic symmetric operations (translation, rotation, mirror, inverse reflection) and the mixing symmetric operations (screw rotation and glide mirror) are accomplished by the local feature extraction and the basic equivariant transformation. According to the quantitative analysis of intermediate data and a large number of test verification, the designs of capsule representation with appropriate chemical environment are demonstrated to be crucial for symmetry recognition and material property predictions.

Supplementary Note 3: The detailed information of datasets

In this work, we achieved the excellent prediction of multiple material properties, benefited from the intelligent description of chemical environment via the SEN model, mainly including the feature extraction (FE), and symmetry perception (SP) blocks.

Consistent with the data source in previous machine learning studies on crystal materials[18-21], the datasets for predicting bandgap and formation energy in this work are sampled from the Materials Project (MP) database. The datasets for bandgap and formation energy contain 6,027 and 30,000 materials, respectively. Both datasets are composed of 64 elements, which cover the entire periodic table except for the noble gases group, lanthanides, actinides, and radioactive elements. Most of these compounds are discovered in experiments, and the remaining ones are predicted by theoretical calculations.

The 30k formation energy dataset is attained by the standard DFT calculations with the PBE exchange-correlation function. The 6k bandgap dataset is obtained by the DFT calculations with the HSE (Heyd-Scuseria-Ernzerh) screened hybrid function, which accounts for the many body effects beyond the single-electron approximation and thus offers more accurate prediction of bandgap than standard DFT calculations. Both datasets contain elemental compositions and multi-element compounds (single element, binary, ternary, quaternary, pentad, and hexaelement crystal materials). The material distributions in different crystal systems of our datasets are shown in the Supplementary

Table 2.

Supplementary Table 2. Material distributions in different crystal systems.

Number	Crystal system	Material
1	Triclinic	8%
2	Monoclinic	12%
3	Orthorhombic	13%
4	Tetragonal	11%
5	Trigonal	14%
6	Hexagonal	16%
7	Cubic	31%

Supplementary Note 4: Detailed explanation of model architecture

The exact feedforward propagation frameworks of the FE and SP blocks are summarized in Supplementary Box 1 and Box 2.

Box 1 | Algorithm 1 Feature extraction block

Input: atom $x_m^{atom} = (a_1, \ldots, a_n) \in M^n$, $x_m^{bond} = (b_1, \ldots, b_k) \in M^k$

Trainable transformer: $\mathcal{F}_c = (\mathcal{F}_c^a, \mathcal{F}_c^b, \mathcal{F}_c^e)$, $SET(\cdot)$, $Att_{Sto}(\cdot)$, $Att_{LSTM}(\cdot)$

Output: $x_m^c = (x_1^c, \ldots, x_m^c) \in M$

$\boldsymbol{v}_i^{atom} \leftarrow \mathcal{F}_c^a(x_i^{atom})$ for all atoms i in input crystal

$\boldsymbol{v}_j^{bond} \leftarrow \mathcal{F}_c^b(x_j^{bond})$ for all bonds j in input crystal

$\boldsymbol{\mathcal{V}}_m^A \leftarrow SET((v_1^{atom}, \ldots, v_i^{atom}) \oplus (v_1^{bond}, \ldots, v_j^{bond}))$ $\forall i, j$

$\boldsymbol{v}_i^{element} \leftarrow \mathcal{F}_c^e(x_i^{atom}, x_{k,k\neq i}^{atom})$ $\forall i, k$

$\boldsymbol{\sigma}_i^{element} \leftarrow Att_{Sto}(x_i^{atom} \odot x_{k,k\neq i}^{atom})$ $\forall i, k$

$\boldsymbol{V}_m^E \leftarrow softmax(v_i^{element} \cdot \sigma_i^{element})$ $\forall i$

$\boldsymbol{x_0} \leftarrow \mathcal{V}_m^A \otimes V_m^E$ $\forall m$

$\boldsymbol{x}_{m,t=0}^c \leftarrow x_0$

For t iterations **do**

  $\boldsymbol{x}_{m,t}^c \leftarrow Att_{LSTM}(x_{m,t}^c)$

End for

$\boldsymbol{x}_m^c \leftarrow x_{m,T}^c$ $\forall m$

Return $\boldsymbol{x}_m^c = (x_1^c, \ldots, x_m^c)$

The input data includes the information of N atoms and M bonds in the primitive cell of target material. The $\mathcal{F}_c$ transformer models possess three different attention-based encoders that produce atomic tensors and bond tensors with (N×256) dimensions.

The SET is a set transformer based on concatenation operation to build atomic chemical environment with ($N \times 192$) dimensions incorporating atom and bond information. The Att_{Sto} is a stoichiometry transformer in terms of an attention-based weight reconstruction model to describe the element correlations. The Att_{LSTM} is a LSTM-based attention model to encode multi-range correlations between atoms.

**Box 2 ┃ Algorithm 2** Symmetry perception block	
**Input:** chemical environment $x_m^c = (x_{m_1}^c, \dots x_{m_n}^c)$	
**Trainable transformer:** $Att_{Conv}(\cdot)$, $F_f^{split}(\cdot)$, $\mathcal{T}_c(\cdot)$, $F_{cap}(\cdot)$	
**Output:** $y_m = (y_1, \dots, y_m) \in M$	
$\boldsymbol{x_m^{Cap}} \leftarrow Att_{Conv}(x_m^c)$	$\forall m$
$\boldsymbol{Cap_m^S}, \boldsymbol{Cap_m^C}, \boldsymbol{Cap_m^P} \leftarrow F_f^{split}(x_m^{Cap})$	
$\boldsymbol{Cap_m^U} \leftarrow \mathcal{T}_c(Cap_m^S, Cap_m^C)$	
$\boldsymbol{\sigma_m^{cap}} \leftarrow Cap_m^U \otimes Cap_m^P$	
$\boldsymbol{Cap_m^{out}} \leftarrow F_{cap}(\sigma_m^{cap})$	
Return $\boldsymbol{Cap_m^{out}} = (Cap_1^{out}, \dots, Cap_m^{out})$	

Herein, the Att_{Conv} is a convolution-based attention transformer that outputs 16 part capsules with (16×128) dimensions to deconstruct the diverse spatial patterns of material. The F_f^{split} is a split transformer that outputs a capsule set (N = 16) of the material via perceiving chemical environment from the part capsules. The $\mathcal{T}_c$ is a symmetry operator that propagates the geometric transformations into the part capsules, mainly including scaling, translation, rotation, inversion reflection, and mirroring reflection transformations.

The SEN adopts a complex deep learning architecture that encompasses the FE and SP blocks as above documented. Accordingly, we construct the likelihood function by describing the feedforward propagation process of these two blocks.

The FE block perceives the input data of atoms and bonds to construct the chemical environment x_m^c of target material m. The corresponding likelihood function takes the form of

$$\mathcal{L}_{FE} = \prod_i^N \prod_j^K \underbrace{P(x_m^c | x_{m,i}^{atom}, x_{m,j}^{bond})}_{\text{Feature extraction}} \qquad (S4)$$

where i is the ith atom in the mth crystal, j is the jth bond in the mth crystal, N is number of atoms in the mth material, K is number of bonds in the mth material.

In the symmetry perception stage, we first construct a sufficient set of material capsules to perceive and to inherit the crystal symmetry based on the material chemical environment, and then establish a non-linear mapping between material capsules and target property. The corresponding likelihood function can be written as

$$\mathcal{L}_{SP} = \prod_{m}^{M} \underbrace{P(y_m | \emptyset_{cap}(x_m^c))}_{\text{Property prediction}} \quad (S5)$$

where *m* is the *m*th crystal in database, *M* is the number of crystals in database, y_m is the ground truth value of property for the *m*th material, $\emptyset_{cap}$ is a set of models in the SP block.

The likelihood function describing the whole feedforward propagation process from material input data to property prediction can be obtained through the production of likelihood functions related to the two subprocess. To make the presentation clearer, we modify the likelihood function as

$$\mathcal{L} = \prod_{m}^{M} \prod_{i}^{N} \prod_{j}^{K} [P(y_m | \emptyset_{cap}(x_m^c)) P(x_m^c | x_{m,i}^{atom}, x_{m,j}^{bond})] \quad (S6)$$

The log likelihood function rather than the likelihood function is more prevalent in the area of machine learning, because the log likelihood function typically illustrates the error evaluation standard. The presentation of likelihood function in our manuscript is intended to reflect the sequential contributions of both FE and SP blocks to property prediction. We now believe that the log likelihood function is equally important, and take the theoretical form as

$$\log(P(y_m)) = \sum_{m}^{M} \sum_{i}^{N} \sum_{j}^{K} \left(\log\left(P(y_m | x_{m,i}^{atom}, x_{m,j}^{bond})\right)\right) \quad (S7)$$

Following, the general framework of the SEN model is illustrated in Supplementary Figure 1.

The feedforward propagation process of the SEN model mainly consists of the FE and the SP blocks. The FE block perceives the input data of atoms and bonds to construct the chemical environment x_m^c of target material following the standard procedure developed by Xie and Grossman[18]. The atom chemical environment of each atom and element weight correlation in primitive cell are built via iterative updating of node-wise computation on atom and bond embedding vectors. The material chemical environment x_m^c of each material is then constructed based on the updated atom tensors, bond tensors, and element weight.

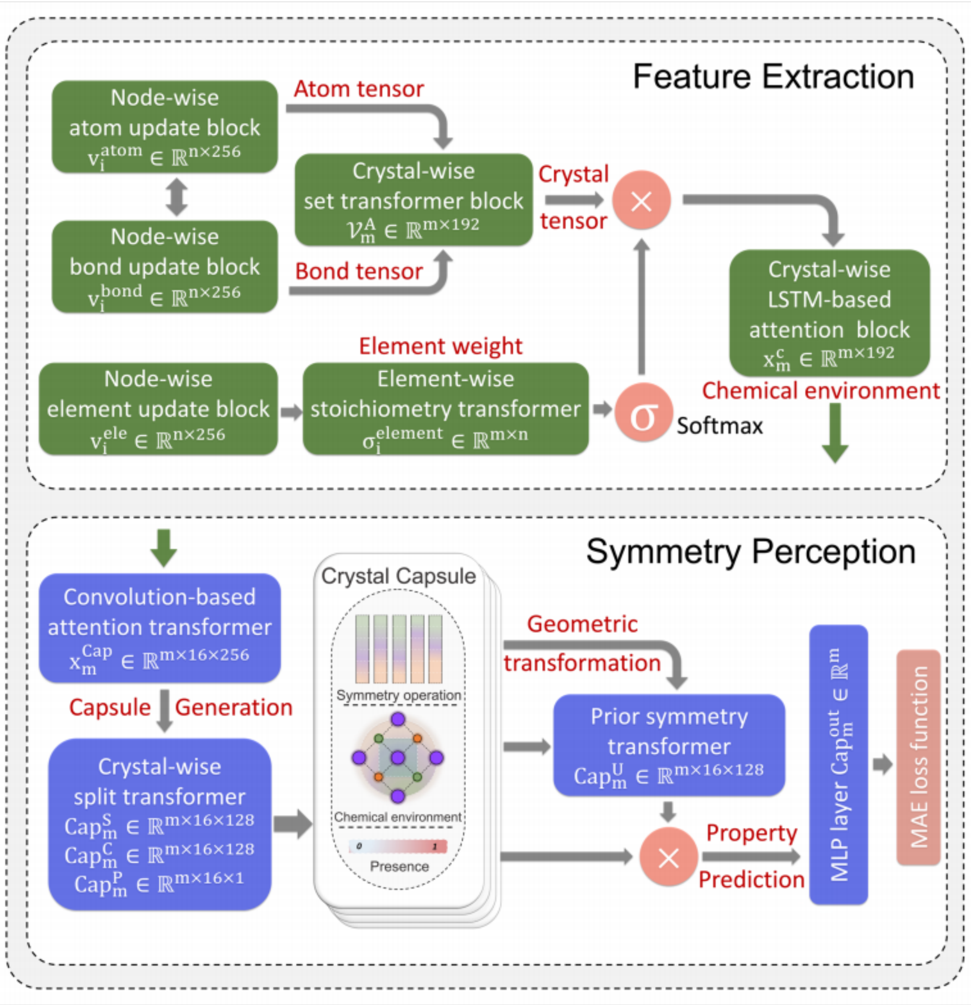

Supplementary Figure 1. Illustration of the SEN architecture. The SEN architecture comprised of the feature extraction, symmetry perception, and property prediction blocks, wherein σ is a SoftMax activation function, the ⊗ stands for multiplication, the LSTM is the long-short term memory model, the MLP is the multilayer perceptron and the MAE is the mean absolute errors.

In the SP block, we construct a sufficient set of material capsules to perceive and to inherit the crystal symmetry after receiving the chemical environment of material. Each capsule is composed of a symmetry operator Cap_m^S, a convoluted material chemical environment Cap_m^C, and a presence value Cap_m^P. Abundant spatial transformation operations will be passed into Cap_m^S through the symmetry transformer, and combined with Cap_m^C to output the updated crystal capsules Cap_u^S. Finally, the target properties are predicted via a MLP-based mapping function, with the input as the multiplication of the capsule presence vectors and updated crystal capsules.

Stacked Capsule Autoencoders (SCAE) describe spatial relationships and similarities between capsules by extracting geometric features of multi-scale patterns[9]. The SCAE model has achieved excellent performance in image classification, reconstruction, and a lot of unsupervised tasks. Our work innovatively applies the capsule mechanism to material property prediction, which has complicated data types

and underlying physical features that are distinct from the graphics applications. Therefore, we establish the FE block and recompile the SCAE as the SP blocks. Detailed schematic diagrams of the atom/bond/element update blocks and SP block are summarized in Supplementary Figure 2.

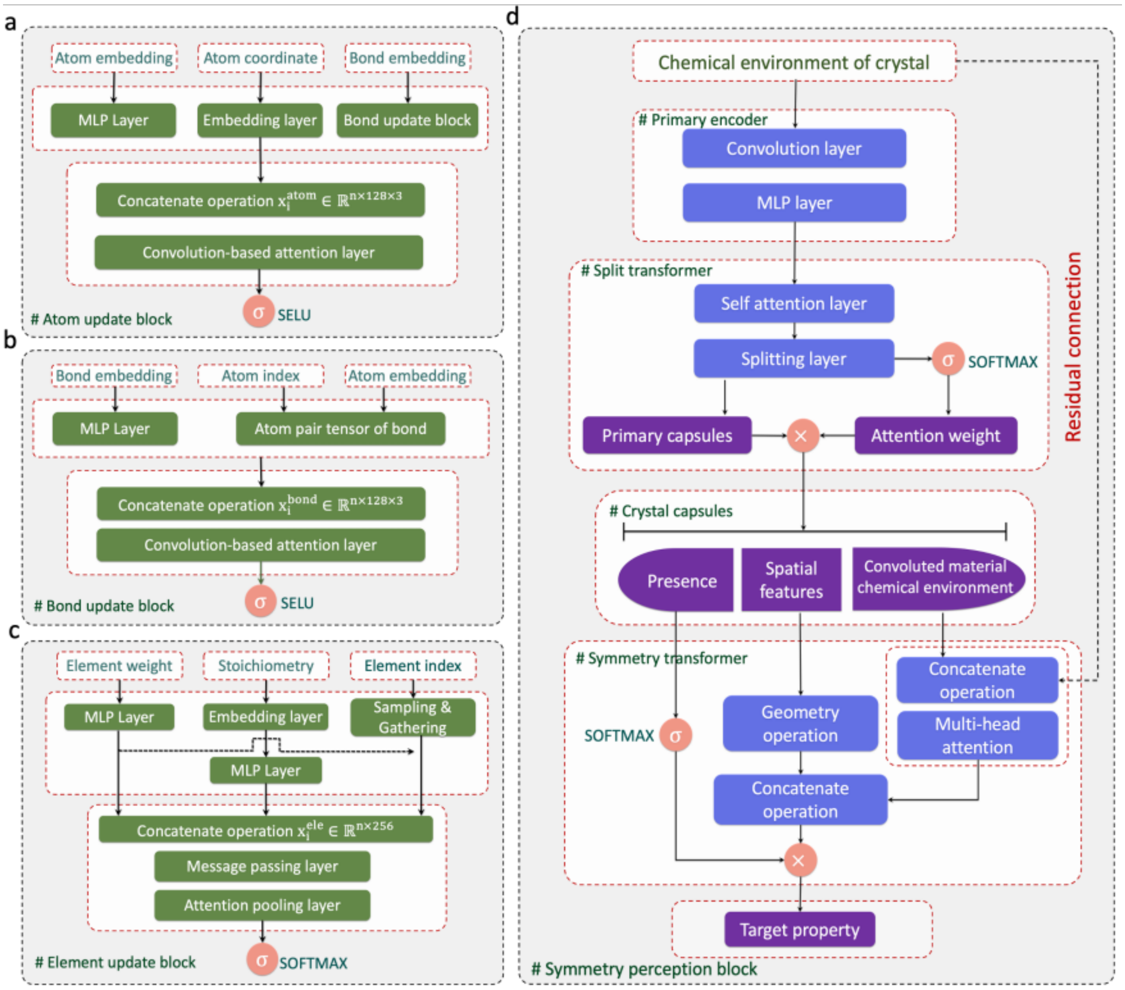

Supplementary Figure 2. Detailed illustration of the SEN model. (a) the atom block simulates the interatomic interaction by receiving atomic information data and performing node-wise calculations, wherein the MLP is the multilayer perceptron. (b) the bond block constructs a crystal graph based on bond index and atom index, and calculates the bond environment. (c) the element update block calculated the element weight for corresponding target property. (d) the SP block in the SEN model is an encoder based on capsule mechanism which is used to encode the prior symmetry transformations and detect equivalent atoms. Wherein σ is a SoftMax activation function, the ⊗ stands for multiplication, the LSTM is the long-short term memory model, and the MAE is the mean absolute errors.

Specifically, all of the MLP layers in the atom, bond, and element update blocks are single full connection layers with 128 nodes and SELU activation functions. The embedding layer also outputs a 128-dimension vectors. The convolution-based attention models in atom and bond update blocks learn the attention coefficients via extracting spatial and chemical features of atomic clusters through convolution and attention mechanisms (Fig. S2 a, b). The convolution-based attention models are composed of a convolution layer with 64 filters and (3×3) kernel sizes, and an attention-

based pooling layer that outputs the updated feature tensors incorporating attention coefficients and chemical environments. The atom pair representation with bond index is employed for bond updating, wherein each bond is denoted by a (2×128) dimensional tensor. In the element update block, the sampling and gathering operations are applied to propagate and to update the element vectors along element index in corresponding crystal. With the input of material stoichiometry and element index, the element weight matrix is calculated by the message passing and attention pooling models as shown in Supplementary Figure 2 c. The message passing layer is an MLP-based graph network generated by the standard procedure developed by Du[23]. The SELU nonlinearity functions are employed at the end of the atom and bond update blocks, while the Softmax function is used at the end of the element update block.

In the SP block, we develop the capsule model for regression prediction of material properties. Both the primary encoder and the geometry operator are implemented in the symmetry perception block for building crystal capsules as illustrated in Supplementary Figure 2 d. Consistent with the standard SCAE, we adopt a convolution-based primary encoder to extract both spatial and chemical features, and to project such information on the primary crystal capsules ($x_m^{Cap} \in \mathbb{R}^{m\times16\times256}$) via a MLP layer with 256 nodes. This convolution-based primary encoder is a series of three convolution layers with 256 filters, (3×3) kernel sizes, and (2×2) strides. A set of crystal capsules are then generated via the split block to divide the information within the primary capsule to three components including the spatial feature Cap_m^S, the convoluted material chemical environment Cap_m^C, and the presence Cap_m^P. In contrast to the straight splitting employed in standard SCAE, an attention-based splitting layer is designed in our SEN model to rationally separating the spatial and chemical information, which is a self-attention layer with 192 neural nodes. The symmetry transformer module is developed to connect the material capsule representation and the material property prediction. In order to fully perceive the chemical environmental information, the residual connection between the Cap_m^C and the material chemical environment is established to update Cap_m^C by the multi-head attention model with 8 heads and 512 neural nodes. Geometric operations associated with the E(n) transformation are then performed on spatial features for updating the Cap_m^S to symmetry operator, which is similar to the operation in standard SCAE. The capsule weight is propagated through the node-wise multiply operation between the capsule presence Cap_m^P and the concatenation of updated Cap_m^C and Cap_m^S. The total amount of parameters in the SEN model are 60K.

Given the difference between the SCAE model[9] and our SEN model in input objects, training purpose, and application scenario, the reconstruction loss cannot be transferred to material property prediction. In general, the complexity of appropriate loss function depends on the complexity of calculation targets, such as property prediction, classification, inverse design, etc. While the image reconstruction task within the SCAE model inevitably demands complicated loss functions, conventional MAE loss is sufficient for our purpose of symmetry identification and single-point

property prediction. This is compliant with the views in material science community that advanced loss functions such as Kullback-Leibler (KL) divergence and Wasserstein distance should only be more suitable for predicting high-dimensional properties such as spectra and temporal series with multi-range correlations. Improper loss function is likely to cause instability, divergence, and even collapse of training. Below we will elaborate on the reasons from the perspectives of theoretical and experimental analyses.

The SCAE model explicitly uses geometric relationships between parts to reason about objects, which segments an image into constituent parts, infers their poses, and reconstructs the image by appropriately arranging affine-transformed part templates. The affine transformation is achieved by performing spatial transformations on each capsule, just like our symmetry transformation. The reconstruction loss of SCAE model is designed to maximize pixel and part log-likelihoods that consist of two parts: a priori loss and a posterior loss. The former is the maximum presence probability among predictions from object capsule k, while the latter is the unnormalized mixing proportion used to explain part capsule m:

$$L = \underbrace{a_k max_m a_{m,k}}_{Prior} + \underbrace{a_k a_{k,m} \mathcal{N}(x_m|m,k)}_{Posterior} \tag{S8}$$

where x_m is the input object, m and k denote the part capsules and object capsules, a_k is the presence probability of capsules, and $a_{k,m}$ is the posterior capsule presence. The prior loss is used to build mapping between different input examples and sets of part-capsules ($a_{m,k}$), and the posterior loss is implemented to specialize object-capsules to particular arrangements of parts ($p(x_m|m,k)$). Spatial transformations within this process have two purposes: the first one is to transmit geometric features into each capsule, and the second one is to reconstruct the object via calculating the geometric correlation.

The reconstruction loss function and training process of SCAE are not suitable for material property prediction because: (1) Image reconstruction mainly depends on the spatial relationship between parts, while material property prediction requires additional physical and chemical features. Reconstruction loss is designed to optimize spatial distribution, and thus cannot assist the learning of intrinsic relationship between the combined spatial, physical, chemical features and the material properties. (2) Geometric transformations in SCAE are designed to simulate spatial relationships between parts and objects, rather than to perceive symmetry features and to identify the equivalence/inequivalence of materials. The reconstruction loss thus cannot improve the training of equivariance identification.

In our SEN model, the material properties are interpreted to depend on the critical atomic clusters and cluster interactions. Therefore, the capsule model is designed to perceive critical equivalent and inequivalent clusters via encoding symmetry transformations, rather than to reconstruct material structural representations. The likelihood function from capsule representation to property prediction is:

$$p(y_m) = \prod_{m=1}^{M} \left[\sum_{k=1}^{K} \emptyset_{cap(p)}^{k} p(y_m | \emptyset_{cap(s)}^{k}, \emptyset_{cap(c)}^{k}) \right] \quad (S9)$$

wherein $\emptyset_{cap(s)}^{k}$, $\emptyset_{cap(c)}^{k}$, and $\emptyset_{cap(p)}^{k}$ are the spatial features, chemical environments, and presence probability in the kth capsule of the mth material. The basic MAE loss function is applied in the training process.

For the implementation of equation (S9), we first constructed material representations based on atomic chemical environments. The cluster representation comprised of multiple crystal capsules, which incorporates chemical and spatial information of atomic clusters, was then initialized by the capsule transformer. Through the operations of symmetry transformation and convolution attention, the crystal capsules were further trained to perceive the equivalence and correlation between clusters. Finally, the weights of capsules were trained by optimizing the likelihood function based on the projection of the correlated crystal capsules on the target attribute (Supplementary Figure 3).

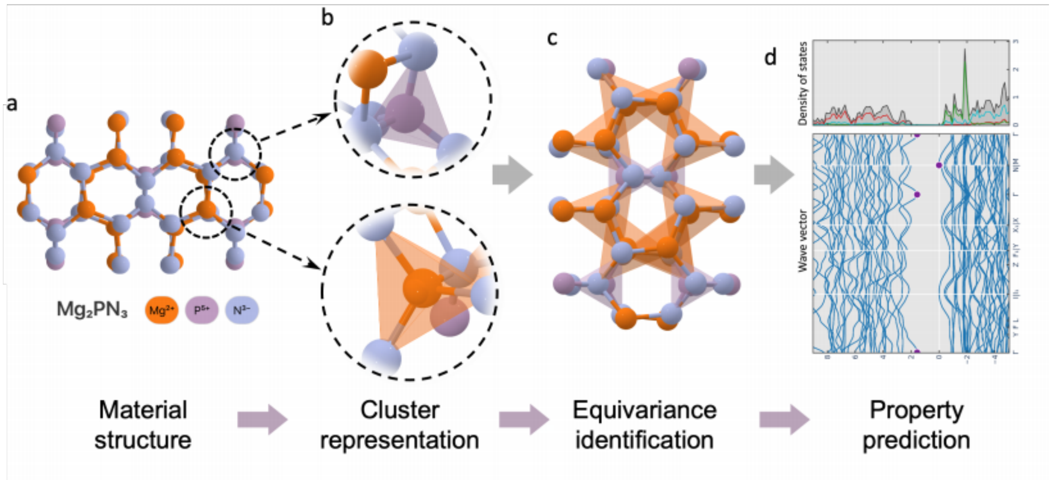

Supplementary Figure 3 Property prediction based on capsule representation, which represents the predictive process form material structure to target property. (a) Material structure of Mg_2PN_3. (b) Build the cluster representation with prior symmetry transformations. (c) Identification of equivalent clusters. (d) Target electronic property, such as bandgap.

The impact of symmetry transformations on model training can be clarified by scrutinizing the feature decoding process of the material capsules. This process mainly consists of equivalent/inequivalent cluster identification and cluster-property projection, which requires the perception of full symmetry transformations and sufficient chemical feature similarities, as schematically illustrated in Supplementary Figure 4. Each crystal capsule is comprised of three components including the spatial feature, chemical environment, and presence probability. The spatial features and chemical environments simultaneously undergo independent feature decoding processes to achieve their own functionalities. Afterwards they work together to achieve cluster equivalence recognition and interaction simulation. Detailed processes can be found in the

symmetry perception block of the workflow as shown in Supplementary Figure 2.

For accurate identification of equivalent/inequivalent clusters, both the spatial equivariance and material chemical information need to be perceived. We therefore built chemical and spatial modules to interpret the components of chemical environments and spatial features in the initial material capsules respectively (Supplementary Figure 4 (b)). The chemical module captured the rich chemical information including atom feature, bond distribution, and stoichiometry to assess the similarity of chemical environments among candidate capsules, and to update the chemical environmental vector of each capsule based on the explored complex correlations. Specifically, the correlation between chemical environments of 16 initial capsules was extracted via the multi-head attention layer.

The spatial module consists of geometry operation, concatenate operation, and convolution-based attention layers. In the geometry operation layer, the initial spatial features of capsules indiscriminately experienced basic spatial transformations through parameterized kernel function of geometric operations. The results for the entire preset transformations (translation, rotation, reflection, mirror, scale, and shear) were concatenated to generate new spatial feature vector for each capsule. The convolution-based attention model was then applied to extract the correlations of these new spatial feature vectors, and to further update the spatial feature vectors of all capsules, which generally activated the spatial transformations that maximize the similarity between spatial features. As identical updated spatial feature vectors (the relevant part of a specific material) would be obtained for equivalent spatial structures, such process accomplished the recognition of spatial equivalence between clusters. Besides, the spatial relationship (distance and alignment) between clusters were implicitly encoded and recorded by the activated spatial transformations.

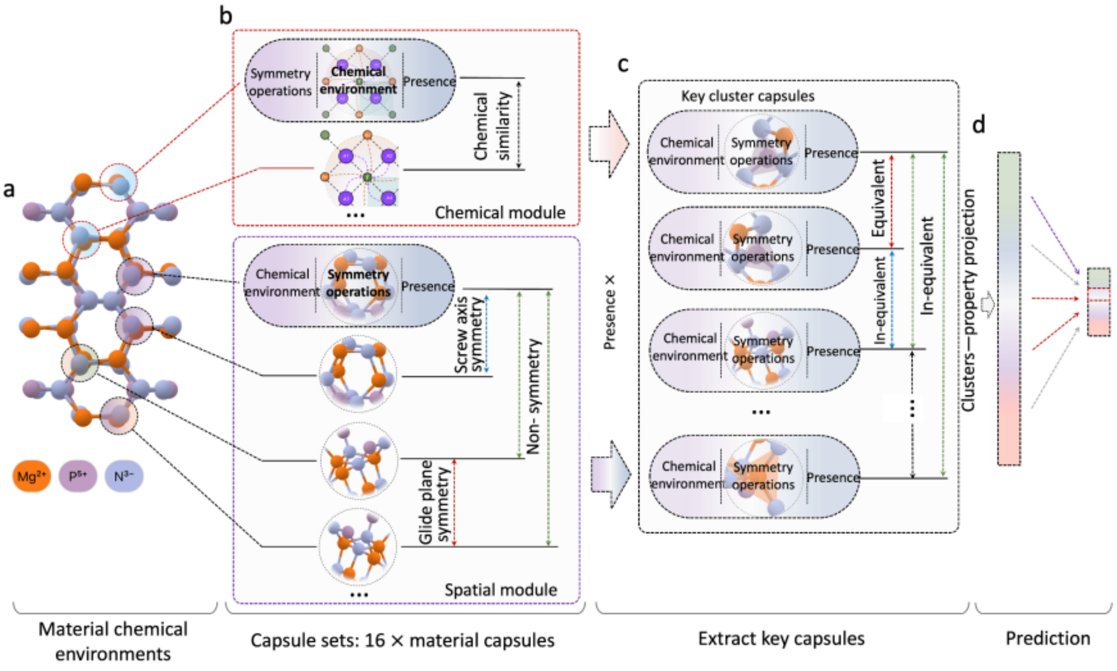

Supplementary Figure 4 Schematic illustration of the training process improved by symmetry transformations. (a) Space groups identification via performing symmetry transformations. (b)

Modular calculation scheme of material capsule characterization mechanism. (c) Extraction of important atom clusters. (d) Property prediction based on the contributions of atom clusters.

Based on the above extraction of feature correlations, the equivalent clusters arising from all crystal symmetries can be accurately identified from the updated cluster capsule representation, because the relevant vector parts of chemical environments and spatial features in the updated capsules associated with equivalent clusters should be identical (Supplementary Figure 4 (c)). To compliant with the perceived correlation between clusters, the presence probabilities of all capsules were updated for appropriately representing the contribution weights of clusters. Finally, the mapping from key cluster contributions to property was established through the simple MLP network (Supplementary Figure 4 (d)). Even though the MLP by itself can not perceive interactions between capsules, our model indeed account for the contribution from cluster interactions to predicted target property, because the capsule correlations obtained by the attention mechanism have been embedded in the updated capsule representation.

It can be inferred from the SEN model framework that the symmetry transformations assist the model training in two aspects. Firstly, the symmetry transformations offer a flexible platform for identifying the equivalence/inequivalence between clusters of each material, as well as for appropriately measuring the similarity between clusters across different materials. Given that equivalent clusters should have identical contributions and similar clusters should have similar contributions to property prediction, the feature space can be substantially reduced and optimized to achieve more precise mapping from clusters to property. Secondly, the symmetry transformations facilitate the perception of relative spatial relationship between clusters, which is crucial to determine the strength of cluster interactions within a material. Through the correlation extraction among capsules based on the attention mechanism, the contributions of cluster interactions can thus be rationally incorporated to improve the property prediction.

Supplementary Note 5: Verification of elemental correlation in crystal obtained by chemical environment.

We calculated the 2-sample Kolmogorov-Smirnov (2-KS) of distinct atomic environments of six materials ($Y_4Cu_2O_7$, $Ca_2Pt_4O_8$, $Ca_2Cu_4O_6$, $KRuO_3$, $Ca_{2/}$ S_2O_8, $Li_2Nb_4O_{11}$) obtained by SEN, which is used to compare the similarity between two distributions, as illustrated in Supplementary Figure 5 a. The 2-KS calculation is accomplished by the SciPy package. The null hypothesis is that the two distributions of atomic chemical environments are identical, and the alternative hypothesis is that the two distributions of atomic chemical environments are not identical. With such hypotheses, the 2-KS value can be applied to evaluate the similarity of atomic chemical

environments. The equivalent atoms should possess very similar distributions, while the inequivalent atoms should possess distinct distributions. The returned quantitative measure is the p-value. All the calculated p-values were less than 0.05, meaning that the null hypothesis can be rejected, which is compliant with the fact that the chemical environment distributions for two different atoms cannot be exactly identified.

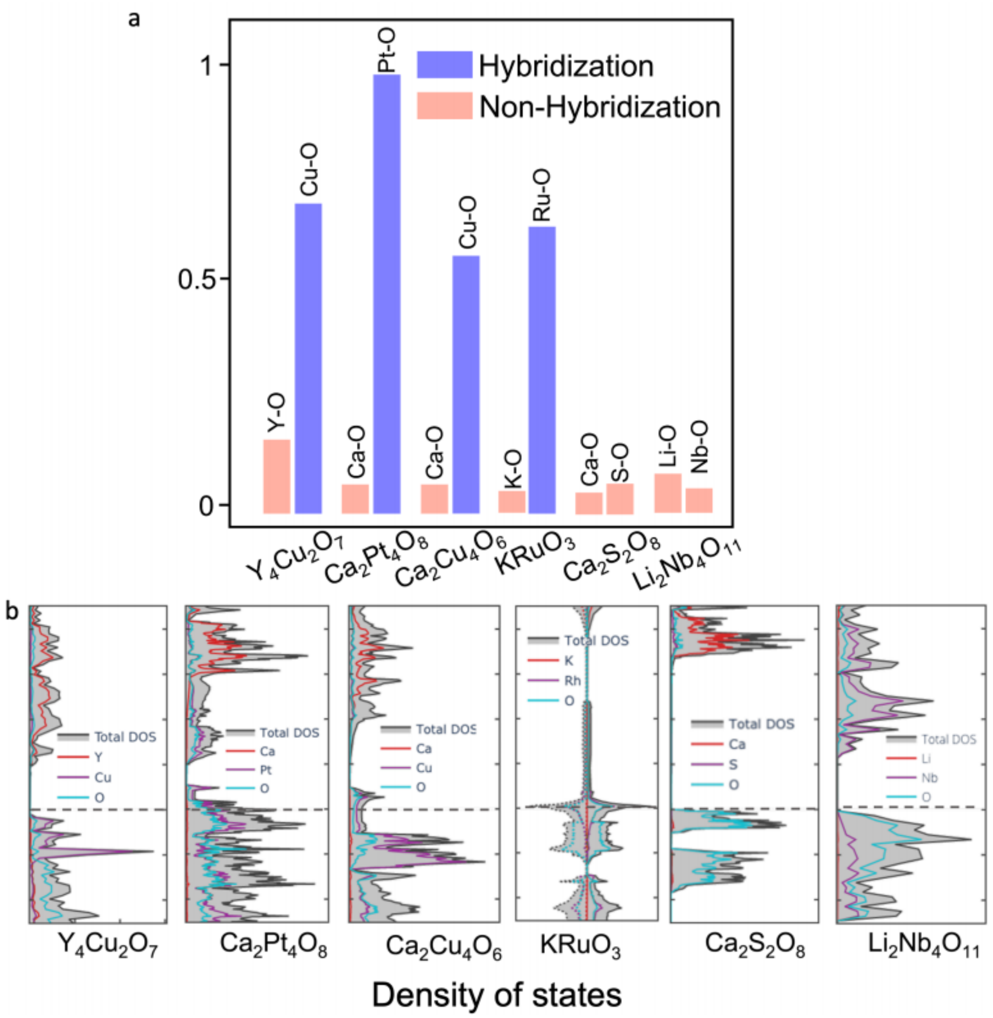

Supplementary Figure 5. 2-sample Kolmogorov-Smirnov a, between two elements of six materials learned by SEN (Symmetry-enhanced equivariance network), and b, corresponding PDOS (partial Density of States) plots obtained by the MP database.

In the histogram plots, the y-axis is the normalized 2-KS value between two atomic chemical environments within the crystals, and red and blue bars indicate whether hybridization occurs in crystals, respectively. The 2-KS results clearly suggest that the hybridization phenomenon in the four materials were successfully detected, which are consistent with their PDOS results, as illustrated in Supplementary Figure 5 b.

The material features successfully encoded by the chemical environment accurately describe the atomic interactions and structural equivalence of crystals. The

6027 crystal materials in the bandgap dataset are composed of 64 elements, which cover the entire periodic table except for the noble gases group, lanthanides, actinides, and radioactive elements. For clear presentation in the main text, 36 elements are selected from all the element groups to reflect the overall trend of the heatmap containing 64 elements. The entire heatmap including 64 elements is shown in Supplementary Figure 6, which completely illustrates the elemental correlations.

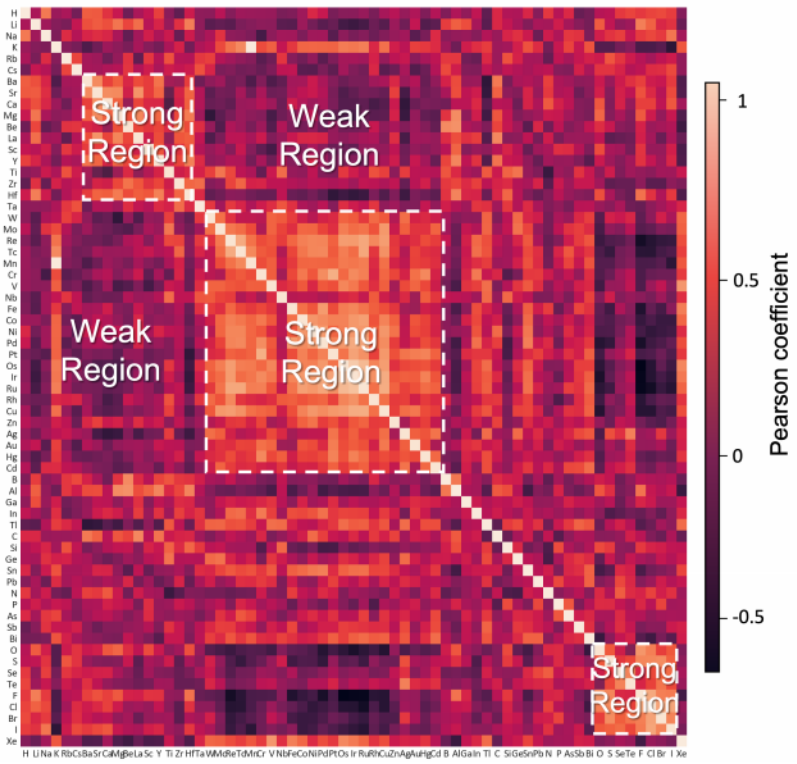

Supplementary Figure 6. Element-based correlation analysis of chemical environments for the entire set of 64 elements obtained by the SEN (Symmetry-enhanced equivariance network) model, with the compounds randomly selected from the MP database and the atoms randomly selected from all atoms within the crystal.

To verify the robustness of elemental correlation map learned from the randomly selected atoms within the crystals, we tested the elemental correlation map of chemical environment for the same set of materials as in Supplementary Figure 6 but with the correlations calculated via weighted averaging on atoms belonging to the same element group (Supplementary Figure 7). Regarding the randomness arising from the selection of compounds, we examined the elemental correlation map of chemical environment for another set of compounds randomly selected from the MP database as illustrated in Supplementary Figure 8. The results suggest that the overall distribution of elemental correlation map is preserved in both test cases, which confirms our conclusions drawn from the map.

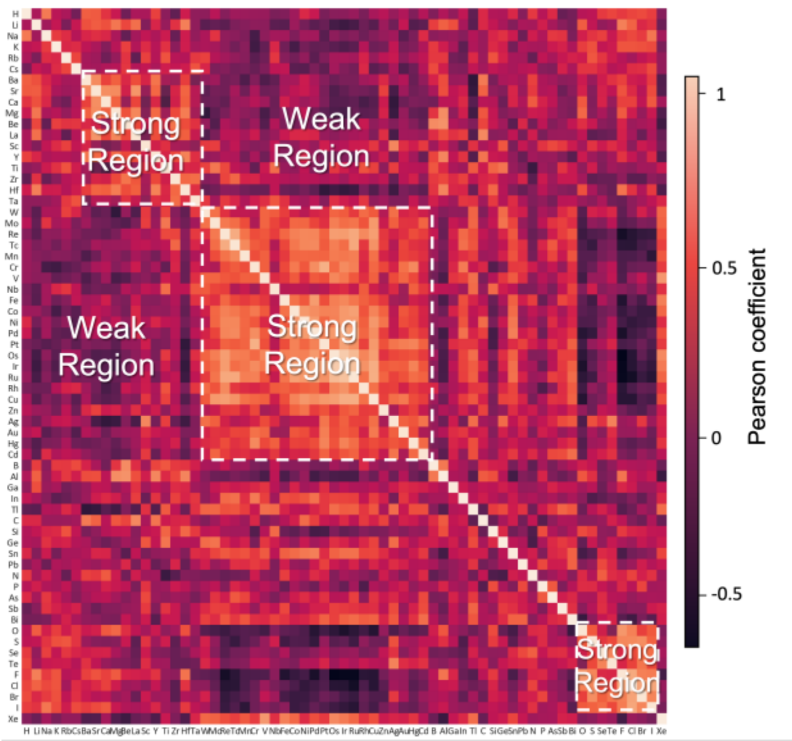

Supplementary Figure 7. Element-based weighted average correlation analysis of chemical environments for the entire set of 64 elements with compounds randomly selected from the MP database obtained by the SEN (Symmetry-enhanced equivariance network) model.

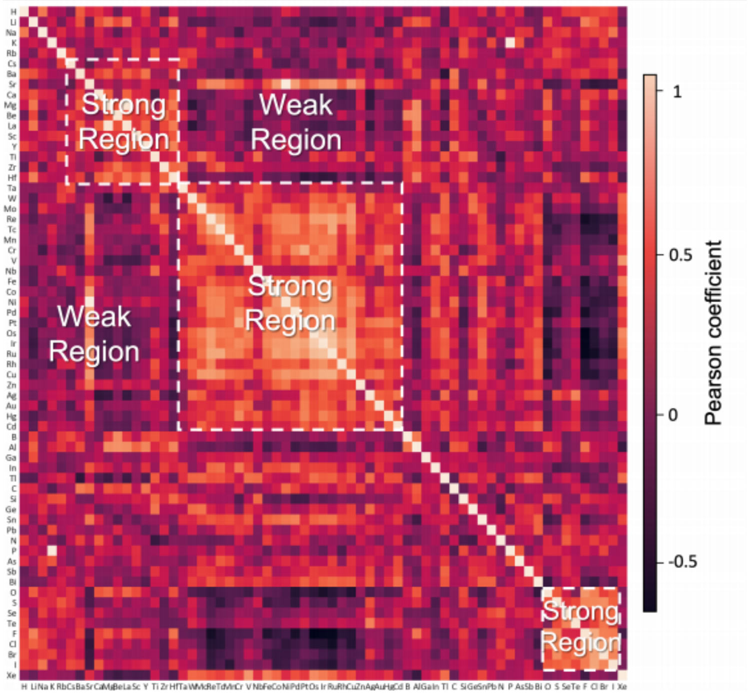

Supplementary Figure 8. Element-based correlation analysis of chemical environment for the entire set of 64 elements with another compound collection randomly selected from the MP database obtained by the SEN (Symmetry-enhanced equivariance network) model.

Supplementary Note 6: Verification of equivariant representation on distinct crystal materials.

In order to quantify the above results, we calculated the 2-sample Kolmogorov-Smirnov (2-KS) of distinct atomic environments in $Y_4Cu_2O_7$ obtained by SEN, as illustrated in Supplementary Figure 9 (left). The y-axis of the histogram plot presents the normalized 2-KS value referring to the same atom Ox. The various colors denote the different types of equivalence between atoms arising from the related to the crystal symmetry (orange: translation, green: rotation, blue: inversion reflection, purple: mirror). Consistent with the original qualitative results, the quantitative KS analysis confirms that all atoms can be correctly divided into a few element groups based on the similar KS values within each group. Almost identical KS values are observed for equivalent atoms, while the distinct KS values appropriately reflect the inequivalent atoms in primitive cell. To further check the robustness of the above analysis, we also calculated the Kullback-Leibler divergence for distinct atomic environments of $Y_4Cu_2O_7$ as shown in Supplementary Figure 7 (right). It turns out that the same conclusions can be arrived through both types of statistical tests.

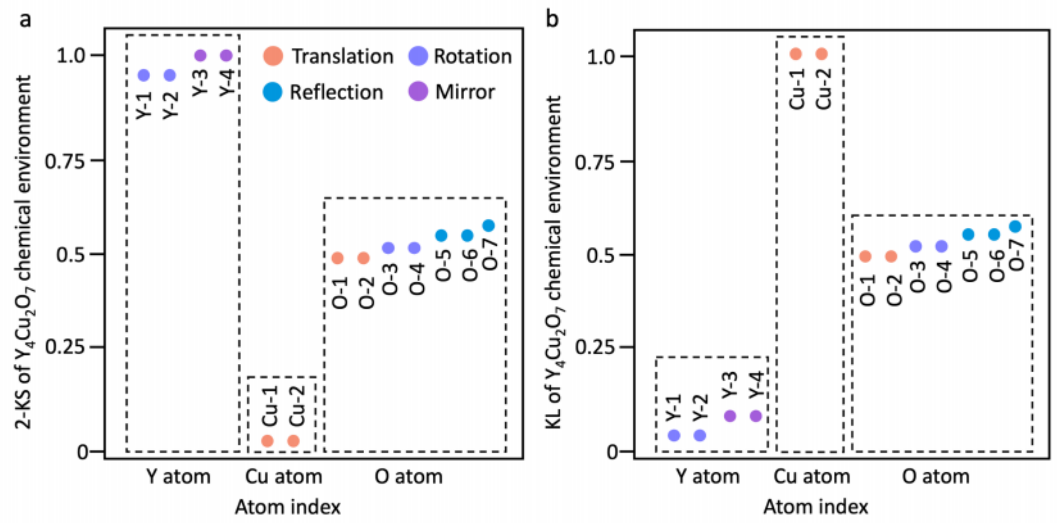

Supplementary Figure 9. (a) 2-sample Kolmogorov-Smirnov and (b) Kullback-Leibler divergence of atomic environments of $Y_4Cu_2O_7$ obtained by SEN (Symmetry-enhanced equivariance network).

In order to quantitatively exam the capability of our SEN to perceive various types of equivalent patterns, we extended the 2-KS calculation to material systems with different symmetric features and different space groups. The equivariance among atoms originating from the translation, rotation, inversion reflection, and mirror symmetry operations are demonstrated by the 2-KS analyses on atomic environments of five materials ($Y_4Cu_2O_7$, $K_4Mo_2O_8$, Li_5MnF_8, $Ca(PtO_2)_2$,

$Li_2Nb_4O_{11}$) as illustrated in Supplementary Figure 10. The complete 2-KS histogram plots of the five materials are further shown in Supplementary Figure 11. Compared with the spatial information obtained from the MP database, it is verified that the SEN model successfully identifies the diverse symmetry transformations and equivalent atomic clusters.

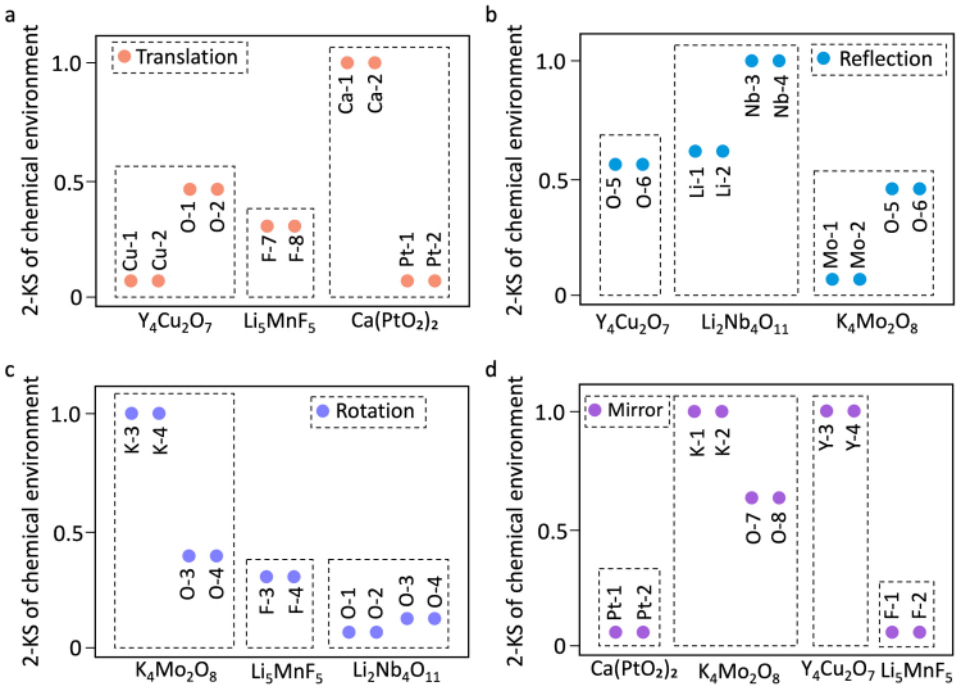

Supplementary Figure 10. The symmetry equivariance perceptions related to four spatial transformations (a) translation, (b) reflection, (c) rotation, and (d) mirror transformations in distinct materials.

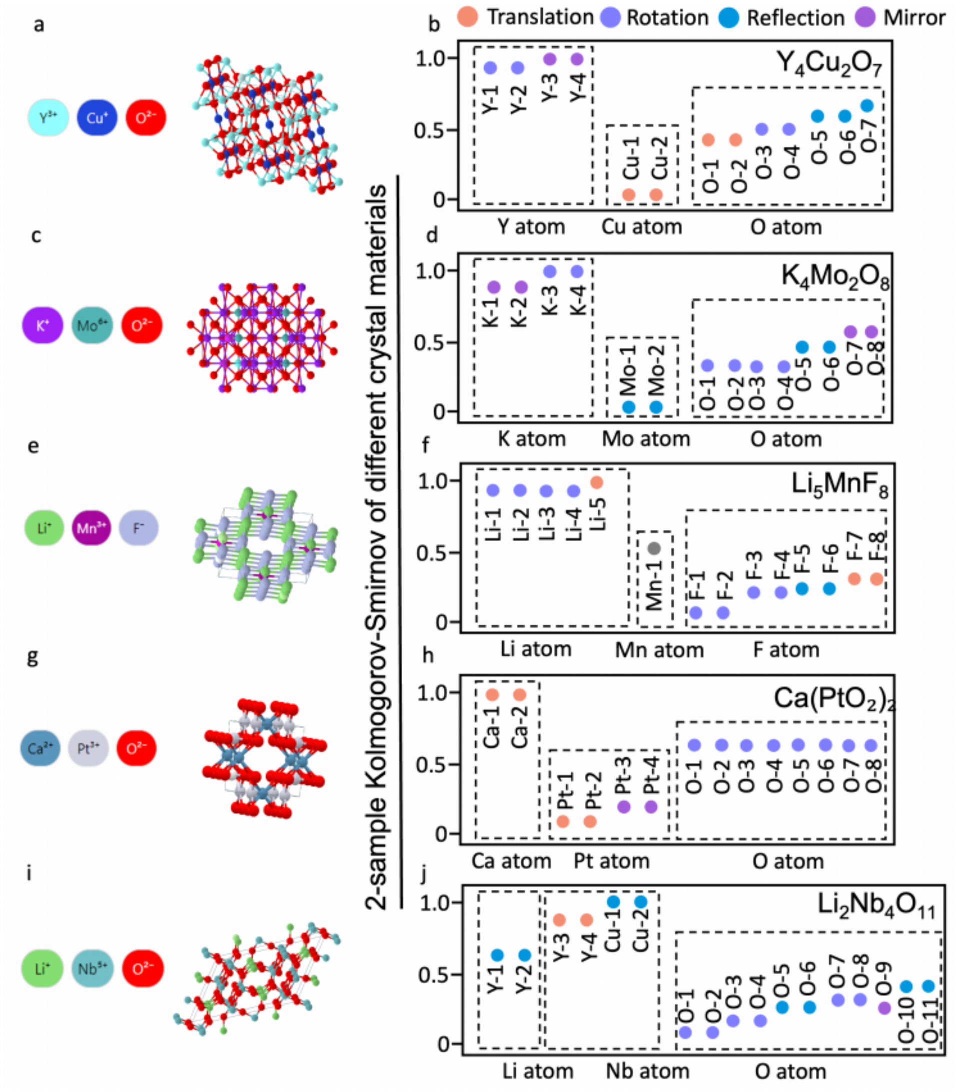

Supplementary Figure 11. The panels (a, c, e, g, j) are corresponding material structures of $Y_4Cu_2O_7$, $K_4Mo_2O_8$, Li_5MnF_8, $Ca(PtO_2)_2$, and $Li_2Nb_4O_{11}$. 2-sample Kolmogorov-Smirnov analyses on atomic environments of (b) $Y_4Cu_2O_7$, (d) $K_4Mo_2O_8$, (f) Li_5MnF_8, (h) $Ca(PtO_2)_2$, (j) $Li_2Nb_4O_{11}$ obtained by the SEN (Symmetry-enhanced equivariance network).

In addition to the original tests on symmorphic transformations, we also sampled six crystal materials that contain features of screw axis and glide plane in space groups. According to the Materials Project database, the equivalent atoms detected by the SEN model are consistent with the crystal symmetries of screw rotation (translation followed by rotation) and glide mirror (translation followed by mirror). The complete 2-KS histogram plots of the six materials containing screw rotation and glide mirror are further shown in Supplementary Figure 12.

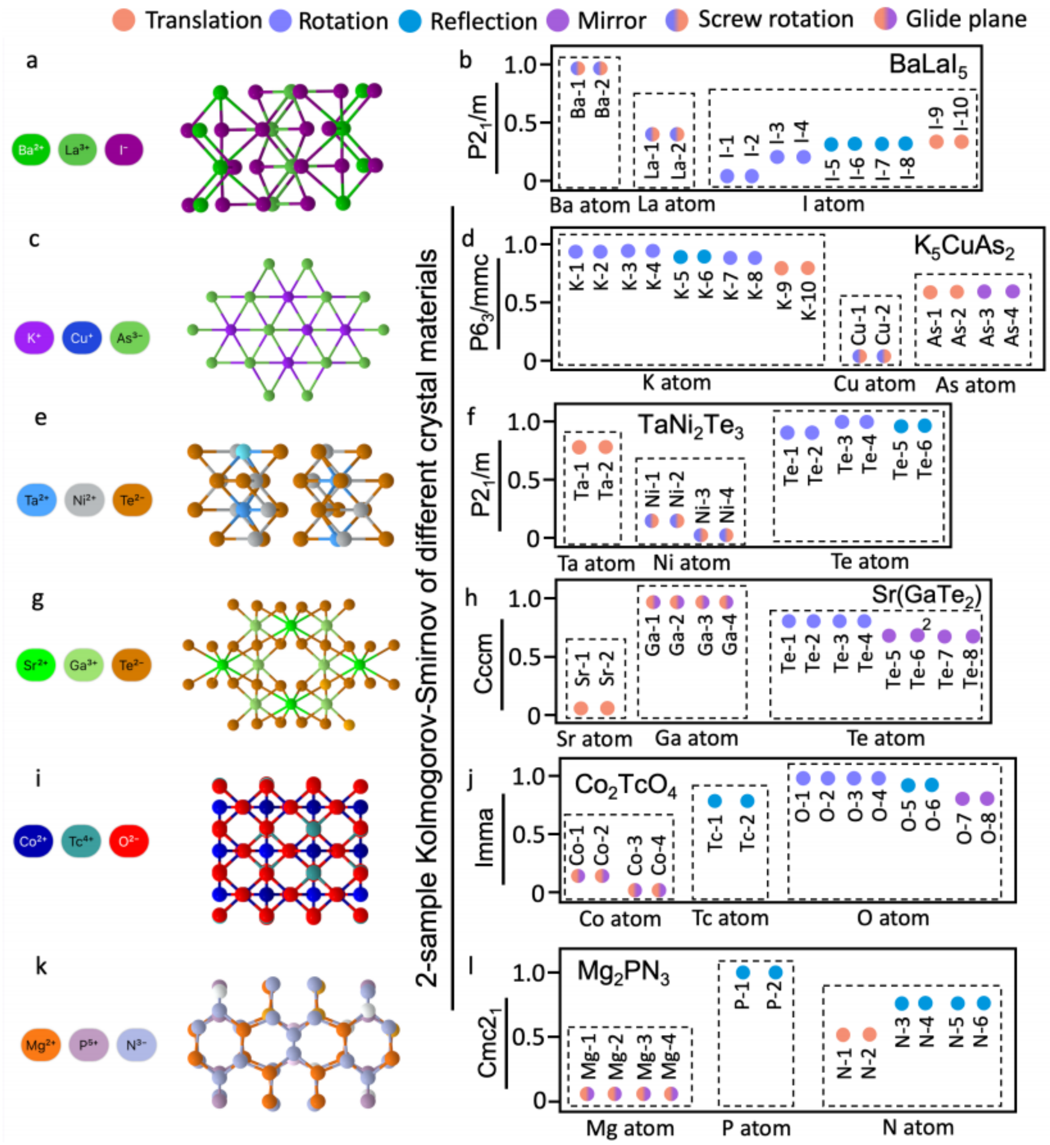

Supplementary Figure 12. 2-sample Kolmogorov-S0mirnov analyses on atomic environments of (a, b) $BaLaI_5$, (c, d) K_5CuAs_2, (e, f) $TaNi_2Te_3$, (g, h) $Sr(GaTe_2)_2$, (i, j) Co_2TcO_4, and (k, l) Mg_2PN_3 obtained by the SEN, wherein panels (a, c, e, g, j, k) are corresponding material structures.

We then explored the mapping from chemical environments to material properties in our SEN model, as shown in Supplementary Figure 13. Five materials were selected from the MP database, including Be_6Ni_2, $Sr_4Ge_2S_8$, $Li_2V_2F_{12}$, $CsAsF_6$, and BaB_2F_8 with the bandgaps of 0 eV, 3.25 eV, 4.86 eV, 7.24 eV, and 10.12 eV, respectively. Strong correlation is observed between the bandgaps and the PDF patterns of material chemical environments, namely the PDF pattern gradually spreads with increasing bandgap.

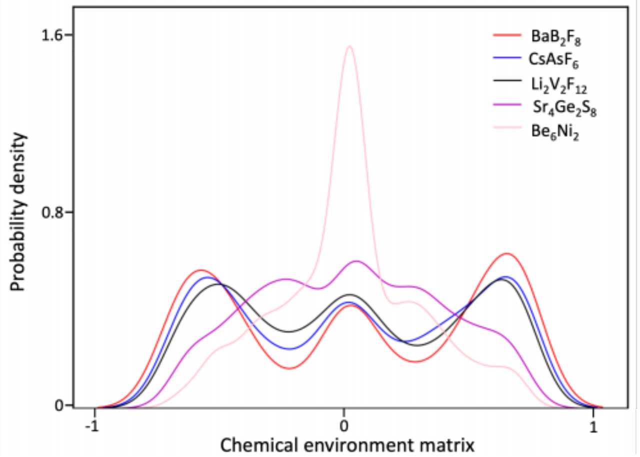

Supplementary Figure 13. Probability density on material chemical environments of samples with various bandgaps, wherein different color lines (red, blue, black, purple, and pink) represent different materials (BaB_2F8, $CsAsF_6$, $Li_2V_2F_{12}$, $Sr_4Ge_2S_8$, and Be_6Ni_2).

To validate the transferability of the SEN model, similar trends have been acquired in the 2D t-SNE plot for formation energy as shown in Supplementary Figure 14.

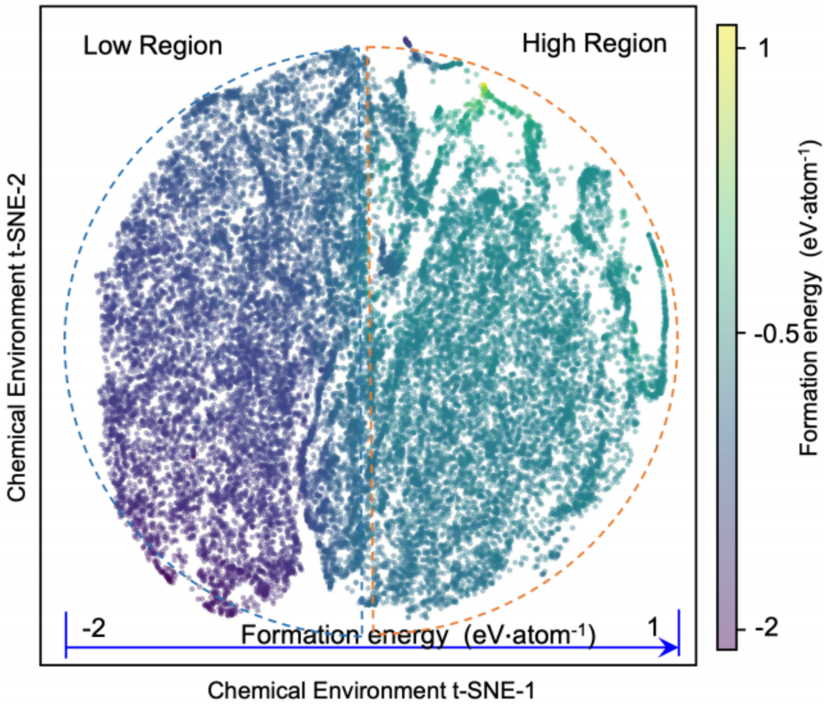

Supplementary Figure 14. The t-SNE plot of chemical environments with 30,000 materials, with the color of circle denoting the formation energy value.

By contrast, the bandgap distributions in the 2D t-SNE plots obtained with MLP and DenseNet models exhibit rough trends but large overlaps between materials with no bandgaps and those with large bandgaps (Supplementary Figures 15-16).

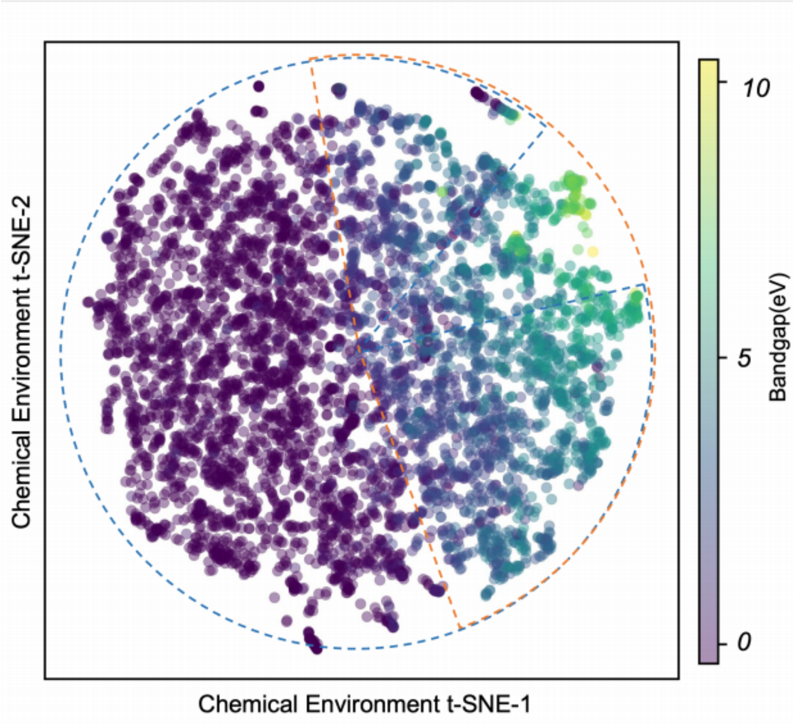

Supplementary Figure 15. The 2D t-SNE plot of chemical environments with 6027 materials, with the color of circle denoting the bandgap value obtained by the SEN with MLP.

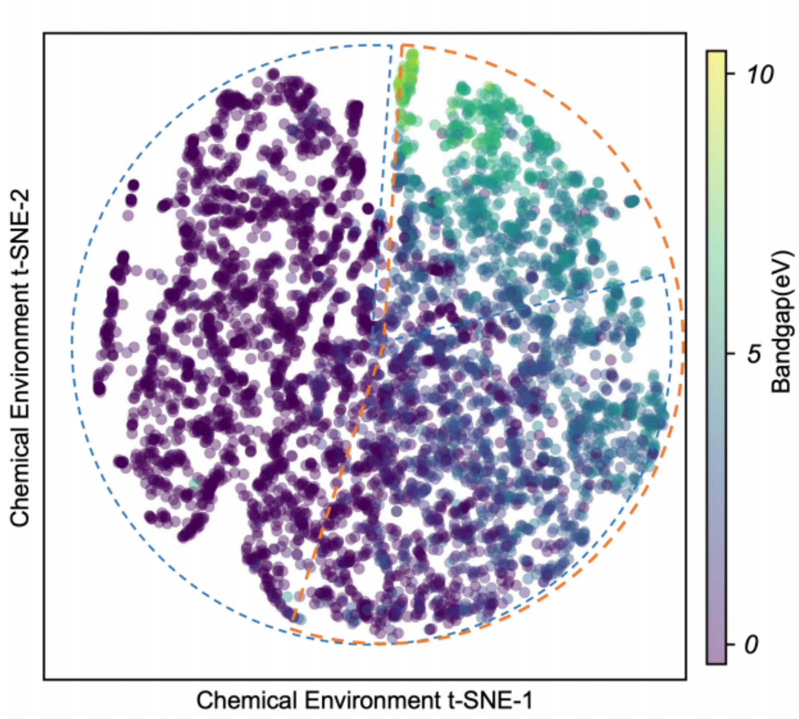

Supplementary Figure 16. The 2D t-SNE plot of chemical environments with 6027 materials, with the color of circle denoting the bandgap value obtained by the SEN with DenseNet.

To demonstrate the capability of our model to encode diverse material symmetries, we investigated the atomic chemical environments in a range of materials comprised of

different elements and within different crystal systems as illustrated in Supplementary Figures 17-18. The results confirm that all equivalent atoms in all of our tested crystals can be clearly identified from the PDF plots of atomic chemical environments.

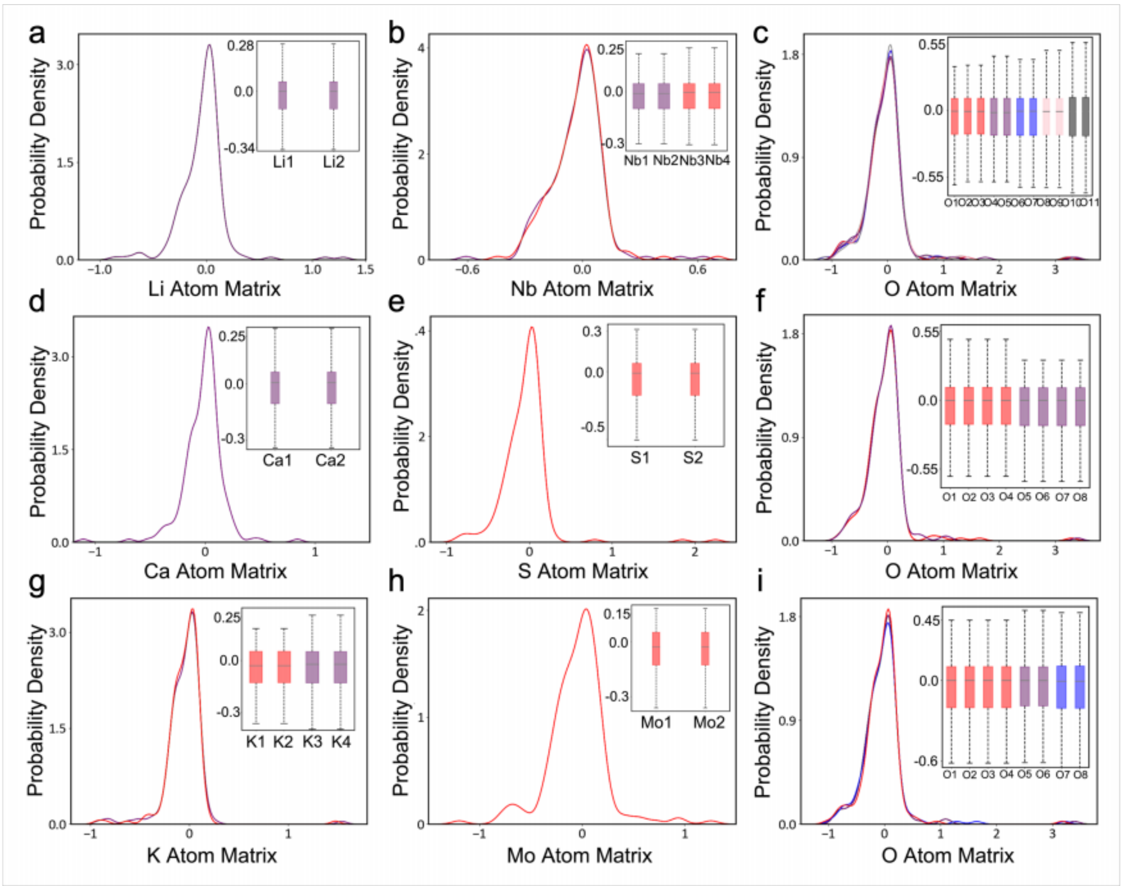

Supplementary Figure 17. a, b, c. PDF on Li, Nb and O atomic environments of $Li_2Nb_4O_{11}$ obtained by SEN, with the associated box plots highlighting the equivariant patterns of different atoms. d, e, f. PDF on Ca, S and O atomic environments of $Ca_2S_3O_8$ obtained by SEN. g, h, i. PDF on K, Mo and O atomic environments of $K_4Mo_2O_8$ obtained by SEN. The box plots show maximum and minimum values (whiskers), upper and lower quartiles (box boundaries) and median values (horizontal lines). The interquartile range, which is the spread between the upper and lower quartiles, covers 50% of the values. The lines with the same color represent different atoms of the same element within the unit cell. The almost identical density distribution patterns among equivalent atoms and their distinctions from those of inequivalent atoms demonstrate the recognition of crystal symmetry.

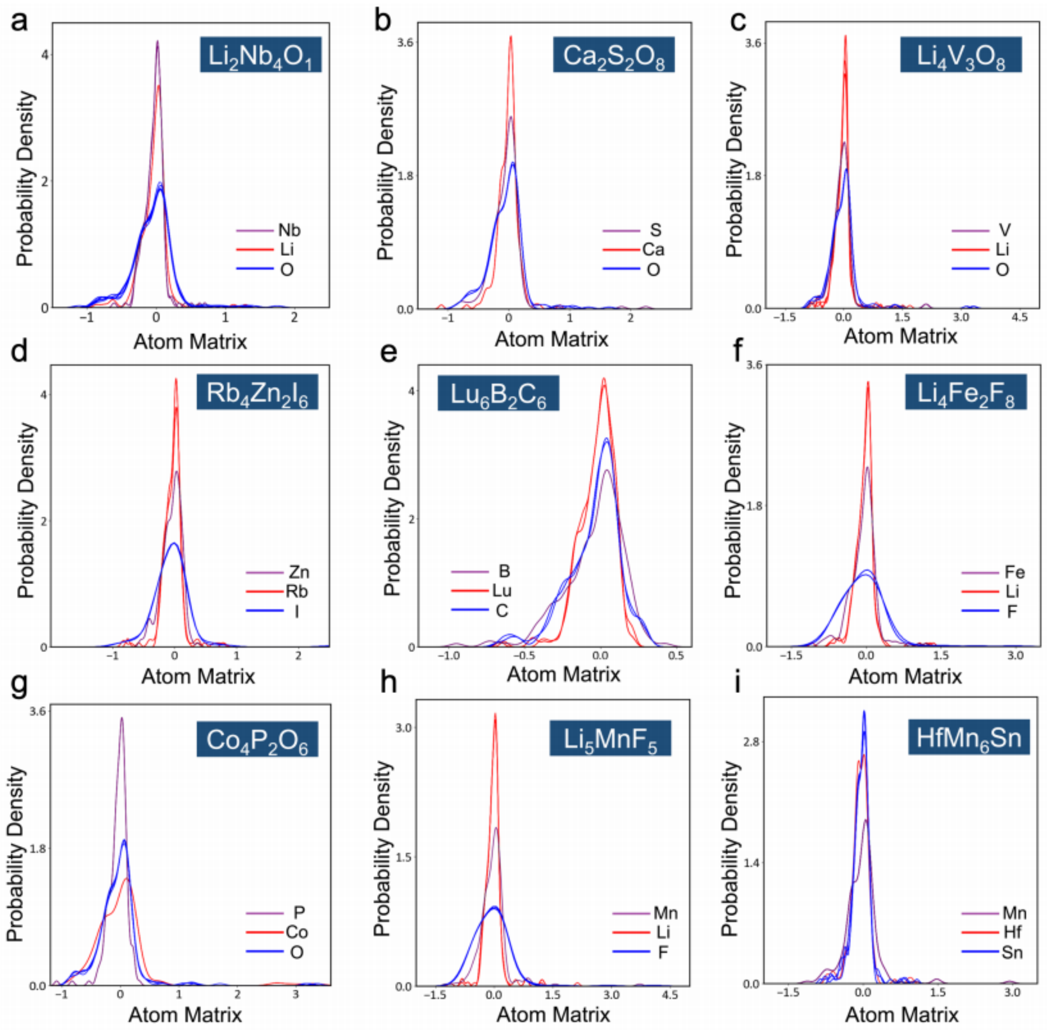

Supplementary Figure 18. Probability densities on atomic environment of (a) $Li_2Nb_4O_{11}$, (b) $Ca_2S_3O_8$, (c) $Li_4V_3O_8$, (d) $Rb_4Zn_2I_6$, (e) $Lu_6B_2C_6$, (f) $Li_4Fe_2F_8$, (g) $Co_4P_2O_6$, (h) Li_5MnF_5, and (i) $HfMn_6Sn_6$ obtained by SEN with capsule layers. The lines with the same color represent different atoms of the same element within the unit cell. The almost identical density distribution patterns among equivalent atoms and their distinctions from those of inequivalent atoms demonstrate the recognition of crystal symmetry.

Further, we perform the performance comparison of perceiving and detecting equivariant features for four models, as shown in Supplementary Figure 19. Consistent with the analysis method of Fig. 3 in the manuscript, we calculated 2-sample Kolmogorov–Smirnov value (2-KS) and probability distribution functions (PDF) for each model. The y-axis of the histogram plot presents the normalized 2-KS values referring to the same atom. Almost identical 2-KS values are observed for equivalent atoms, while the distinct 2-KS values appropriately reflect the inequivalent atoms in primitive cell. We can see that the SEN model can accurate distinct three elements of $Y_4Cu_2O_7$ crystal, and successfully detect the inequivalent and equivalent atoms in crystal. However, in the SEN-TFN and SEN-SE(3) models, the PDF results clearly

forms three curves, which just can identify different elements of $Y_4Cu_2O_7$ crystal, but failed to detect the equivalent atoms due to cannot perceive sufficient symmetry group features. For the SEN-EGNN model, both 2-KS and PDF results demonstrate disorderly pattern, it is hardly to distinct different elements and identify equivariant atoms. This maybe because that the coordinate embedding method in this work is not sufficient to construct a reasonable chemical environment and transfer equivariant spatial features.

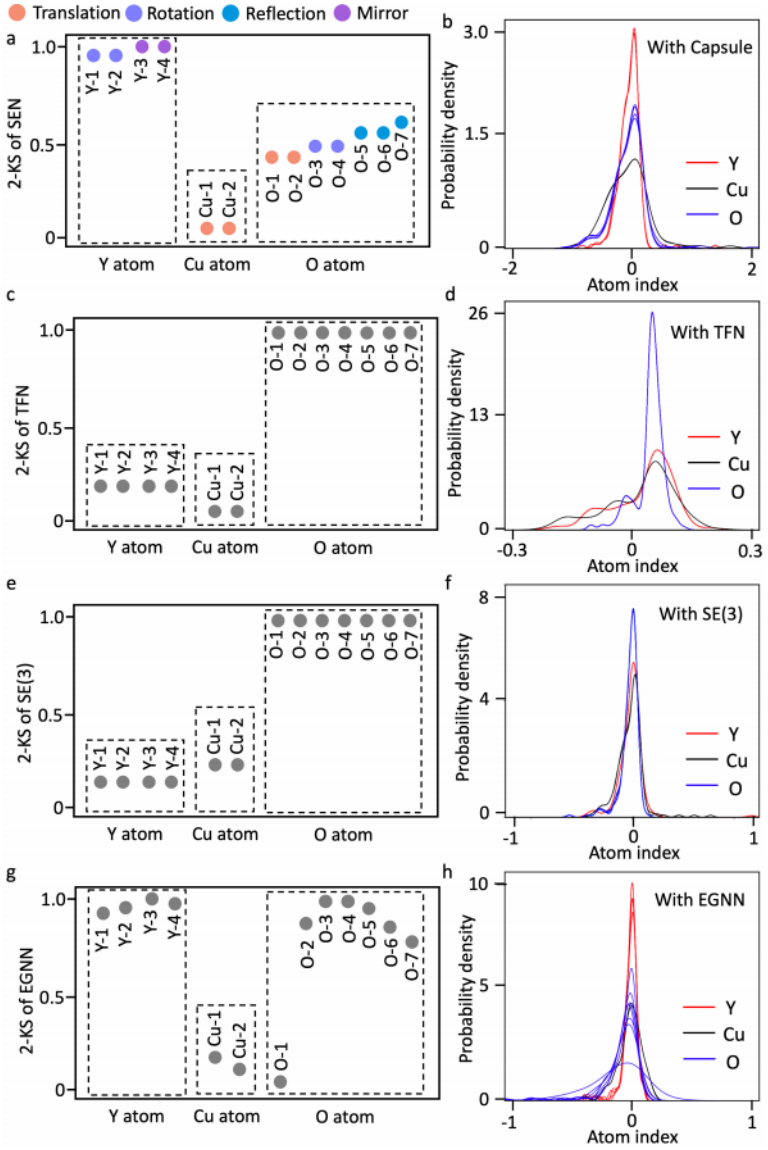

Supplementary Figure 19 The identification verification of equivariant features for (a) the SEN model, (c) the SEN-TFN model, (e) the SEN-SE(3)-Transformer model, and (g) the SEN-EGNN model. The panels (b, d, f, and h) are probability densities obtained by the above models. The lines with the same color represent different atoms of the same element within the unit cell. The almost identical density distribution patterns among equivalent atoms and their distinctions from those of inequivalent atoms demonstrate the recognition of crystal symmetry (panel b). Incomplete learning of crystal symmetry and atomic equivalence lead to chaotic distribution patterns (panels d, f, and h).

We created a table (Supplementary Table 3) to quantitatively present the main results in the figures, and inserted relevant descriptions in the main manuscript. Specifically, the crystal system, space group, bandgap, and normalized 2-KS values of atomic chemical environments are documented as follows.

Supplementary Table 3 The crystal information and the 2-KS values of atomic chemical environments for representative crystal materials.

N	Material	Band gap(eV)	Crystal system	Space group	Atom	Norm KS value
1	$Y_4Cu_2O_7$	1.57	Monoclinic	C2/m	Y	0.98, 0.98; 1, 1
					Cu	0.0033, 0.0031
					O	0.49, 0.49; 0.54, 0.59; 0.58, 0.58, 0.61
2	$K_4Mo_2O_8$	4.07	Monoclinic	C2/m	K	0.89, 0.89; 1, 1;
					Mo	0.0029, 0.0029
					O	0.41, 0.41, 0.41, 0.41; 0.52, 0.52; 0.58, 0.58;
3	Li_5MnF_8	1.98	Orthorhombic	Cmmm	Li	0.92, 0.92, 0.92, 0.92, 1
					Mn	0.55
					F	0.0031, 0. 0031; 0.28, 0.28, 0. 28, 0. 28; 0.42, 0.42;
4	$Ca(PtO_2)_2$	0	Tetragonal	$P4_2/mmc$	Ca	1, 1;
					Pt	0.0035, 0.0033; 0.22, 0.22;
					O	0.73
5	$Li_2Nb_4O_{11}$	2.32	Monoclinic	C2/m	Li	0.68, 0.68;
					Nb	0.81, 0.81; 0.995, 1;
					O	0.0029, 0.0029; 0.18, 0.18; 0.26, 0.26; 0.31, 0.31; 0.35, 0.35; 0.29; 0.42, 0.42;
6	$BaLaI_5$	2.24	Monoclinic	$P2_1/m$	Ba	1, 1;
					La	0.43, 0.43;
					I	0.0018, 0.0018; 0.149, 0.149; 0.34, 0.34, 0.34, 0.34; 0.38, 0.38;
7	K_5CuAs_2	0.25	Hexagonal	$P6_3/mmc$	K	1, 1, 1, 1; 0.97, 0.97, 0.97; 0.97; 0.90, 0.90;
					Cu	0.0015, 0.0015;
					As	0.84, 0.84, 0.84, 0.84;
8	$TaNi_2Te_3$	0	Monoclinic	$P2_1/m$	Ta	0.79, 0.79;
					Ni	0.13, 0.13; 0.0023, 0.0023
					Te	0.93, 0.93; 1, 1; 0.98, 0.98;
9	$Sr(GaTe_2)_2$	0.45	Orthorhombic	Cccm	Sr	0.0017, 0.0017;
					Ga	1, 1, 1, 1;
					Te	0.83, 0.83, 0.83, 0.83; 0.76, 0.76, 0.76, 0.76;
10	Co_2TcO_4	0	Orthorhombic	Imma	Co	0.12, 0.12; 0.0021, 0.0021;
					Tc	0.80, 0.80;
					O	1, 1, 1, 1; 0.90, 0.90, 0.85, 0.85;
11	Mg_2PN_3	3.8	Orthorhombic	$Cmc2_1$	Mg	0.12, 0.12, 0.12, 0.12;
					P	1, 1;
					N	0.55, 0.55; 0.73, 0.73;

Supplementary Note 7: Interpretable verification of chemical environment of crystal obtained by the SEN.

We found that material bandgap depends on complex features and can not be simply predicted by any critical factors by investigating a variety of material features, including component, structure, coordination, bond, point group, crystal group, spin polarization, orbital hybridization (as documented in Supplementary Table 4).

Supplementary Table 4. Material information of Ca-O-X group obtained by Pymatgen in the Materials Project.

Group	Material	Point group	Crystal system	Spin	Hybridization	Bandgap (eV)
Group 1	$Ca_2As_2O_7$	2/m	monoclinic	same	No	4.99
	$Ca_2Y_4O_8$	mmm	orthorhombic	same	No	4.67
	Ca_3CdO_4	mmm	orthorhombic	same	No	3.78
	$Ca_4Sn_2O_8$	mmm	orthorhombic	same	No	4.00
	$Ca_4Hf_2O_8$	mmm	orthorhombic	same	No	5.49
	Ca_2O_2	6/mmm	hexagonal	same	No	4.43
	$Ca_2W_2O_8$	4/m	tetragonal	same	No	5.37
	$Ca_2Ti_2O_6$	4/mmm	tetragonal	same	No	3.75
	$Ca_2Mo_2O_8$	4/m	tetragonal	same	No	4.634
	$Ca_2Si_2O_6$	4/mmm	tetragonal	same	No	5.56
	$Ca_2Y_4O_8$	$m\bar{3}m$	cubic	same	No	4.31
	$CaZrO_3$	$m\bar{3}m$	cubic	same	No	4.77
	CaO	$m\bar{3}m$	cubic	same	No	5.29
	Ca_6HfO_8	$m\bar{3}m$	cubic	same	No	5.89
	$Ca_3V_2O_8$	3m	trigonal	same	No	4.76
	$Ca_2Sn_2O_6$	$\bar{3}$	trigonal	same	No	4.23
	$CaAs_2O_6$	$\bar{3}m$	trigonal	same	No	4.81
	$Ca_2Ti_2O_6$	$\bar{3}$	trigonal	same	No	5.32
	$CaHgO_2$	$\bar{3}m$	trigonal	same	No	3.64
Group 2	CaH_2O_2	$\bar{3}m$	trigonal	same	No	5.75
	$Ca_2C_2O_6$	$\bar{3}m$	trigonal	same	No	6.96
	$Ca_3P_2O_8$	$\bar{3}m$	trigonal	same	No	7.20
	$Ca_2Al_4O_8$	2/m	monoclinic	same	No	6.54
	$Ca_2C_2O_6$	mmm	orthorhombic	same	No	6.19
	$Ca_2C_2O_6$	222	orthorhombic	same	No	7.61
	$Ca_2S_2O_8$	mmm	orthorhombic	same	No	7.67
Group 3	$Ca_2Cu_4O_6$	mmm	orthorhombic	same	Yes	0.00
	$Ca_4Mn_4O_{10}$	mmm	orthorhombic	dif	Yes	1.09
	$Ca_2Co_1O_3$	2/m	monoclinic	dif	No	0.00

	$CaCuO_2$	4/mmm	tetragonal	same	Yes	0.00
	$Ca_2Pt_4O_8$	4/mmm	tetragonal	same	Yes	0.00
	$CaBeO_3$	$m\bar{3}m$	cubic	dif	No	0.00
	$Ca_2Pd_6O_8$	$m\bar{3}m$	cubic	same	Yes	0.11
	Ca_3PbO	$m\bar{3}m$	cubic	same	Yes	0.81
	$Ca_1B_1O_3$	$m\bar{3}m$	cubic	same	No	0.00
Group 4	$CaSnO_3$	$m\bar{3}m$	cubic	dif	No	0.00
	$CsCaO_3$	$m\bar{3}m$	cubic	dif	No	0.00
	$RbCaO_3$	$m\bar{3}m$	cubic	dif	No	0.00
	$KCaO_3$	$m\bar{3}m$	cubic	dif	No	0.00
	$SrCaO_3$	$m\bar{3}m$	cubic	dif	No	0.00
	$LiCaO_3$	$m\bar{3}m$	cubic	dif	No	0.00

'same' is the spin polarization, and 'dif' is the spin splitting. '0' and '1' are non-occurrence and occurrence of hybridization phenomenon between atoms near Femi surface respectively.

We sampled six material groups (Ca-O-X, Al-O-X, Ba-O-X, K-O-X, Sr-O-X, and Li-O-X) from the bandgap data set (with 6,027 crystal materials), which are comprised of binary and ternary compounds in the form of A-B-X with various stoichiometry. The symbols A and B indicate different fix elements, and X is a varying element with its name shown in the figures. The 'A' element contains two alkali metals of the first main group, three alkaline earth metals of the second main group and aluminum elements of the IIIA group. The 'B' element is the oxygen element. The 'X' element is mainly composed of metal elements, alkali metal elements, halogen elements, and some non-metal elements, spanning all main groups except the noble gas. The six material groups contain 408 crystal materials and 42 distinct elements in total.

Nevertheless, rational and unified trends can be detected according to the reduction dimensionality analysis of chemical environments in different material groups as shown in Supplementary Figures 20 (1-3).

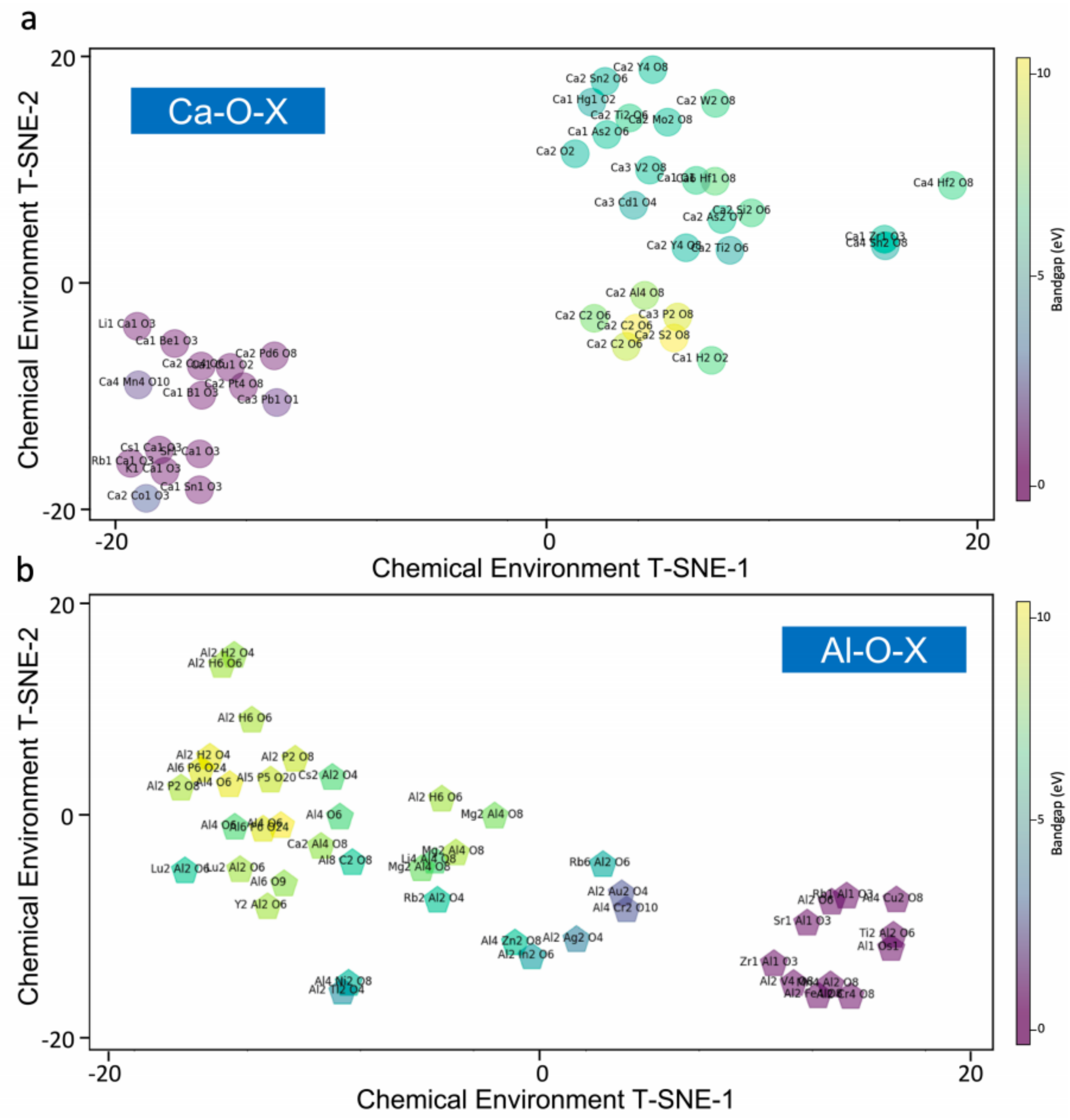

Supplementary Figure 20-1. a-b. Clustering analysis on the chemical environments of Ca-O-X and Al-O-X compounds.

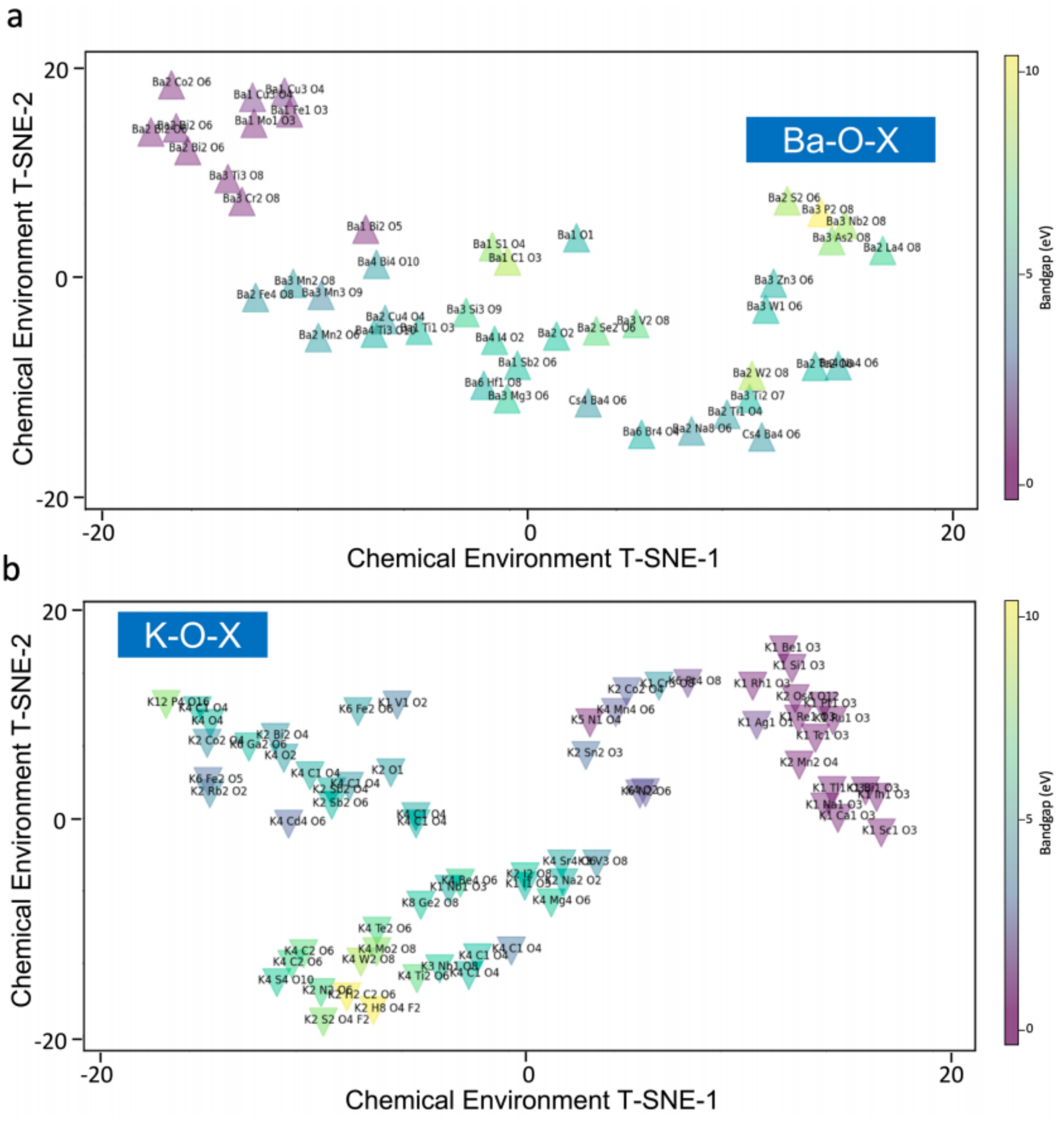

Supplementary Figure 20-2. a-b. Clustering analysis on the chemical environments of Ba-O-X and K-O-X compounds.

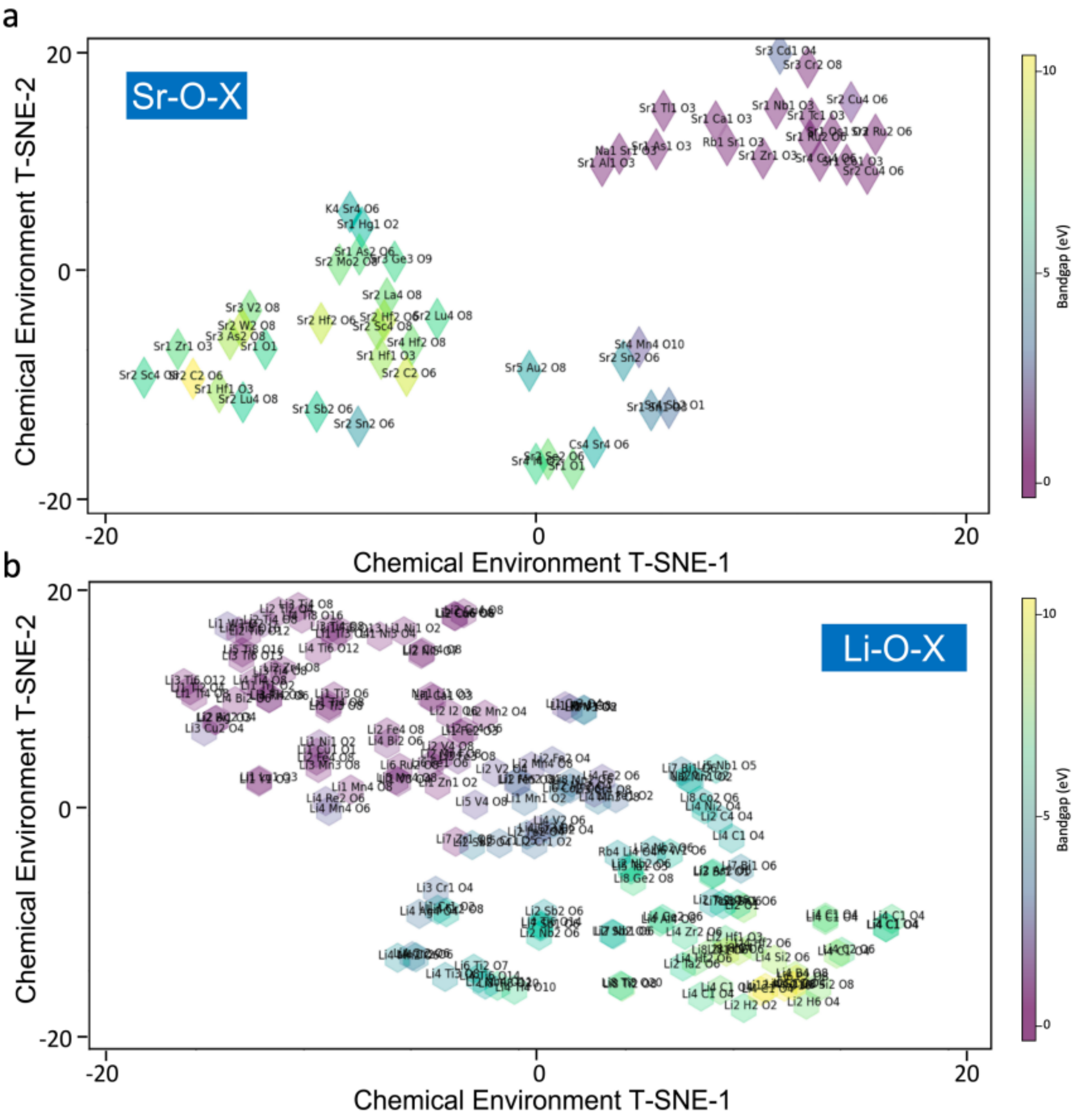

Supplementary Figure 20-3. a-b. Clustering analysis on the chemical environments of Sr-O-X and Li-O-X compounds.

Supplementary Note 8 The interpretation for test experiments

Note that some materials belonged to the trigonal and cubic crystal systems exhibit large error in bandgap prediction by our model, indicating that some important factors are still missing in our material representation. To understand the potential error sources, we scrutinized the electronic and structural features of the materials with the five largest prediction errors. All of them are actually metals but have been incorrectly predicted to be semiconductors. Specifically, $CoRe_2O_8$ and $KCrF_3$ contain magnetic elements and exhibit noticeable spin splitting. The remaining crystals ($LiBeO_3$, $Ba_3Re_2O_9$ and Ba_2LiOsO_6) are nonstoichiometric compounds with unusual bond orders. The incorporation of material features to describe spin and nonstoichiometric environment

is beyond the scope of this study and worth future investigation.

In order to fairly compare the performance among the SEN model, the pre-existing non-equivariant models, and E(n)-based models, we conducted comparative experimental tests on predicting bandgaps. With the preservation of the initial block that establishes the material chemical environments, the capsule block in our SEN model is separately replaced by the relevant blocks of a non-equivariant model (DenseNet) and three representative E(n)-based models including the TFN[15], the SE(3)-Transformer[6], and the EGNN[16] models. The training and testing of different models are carried out the same data set and cross-validation scheme.

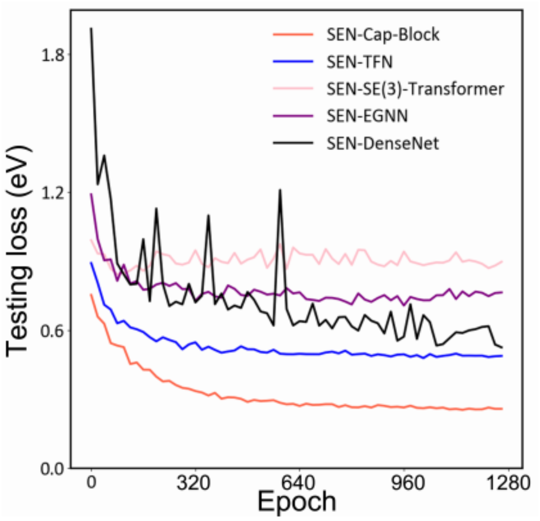

Supplementary Figure 21 The comparison of the testing processes between the SEN model and the pre-existing equivariant/not-equivariant methods.

As can be seen from the Supplementary Figure 21, the SEN model converges to the best prediction error both in the training and testing process. The other three models achieve the convergence effect of training, but are far from achieving the prediction performance acceptable to the physical system. Specifically, the predictive performance rankings of the three models are: (1), SEN-SE(3)-Transformer, (2) SEN-EGNN, (3), SEN-TFN, such results are also consistent with key designs in the three models. And we then extract the bandgap prediction results of the four models after training to 1280 epochs as shown in the Supplementary Figure 22. Both in the training and testing process, the replaced models are difficult to achieve accurate prediction and hard to handle crystals with diverse symmetry groups.

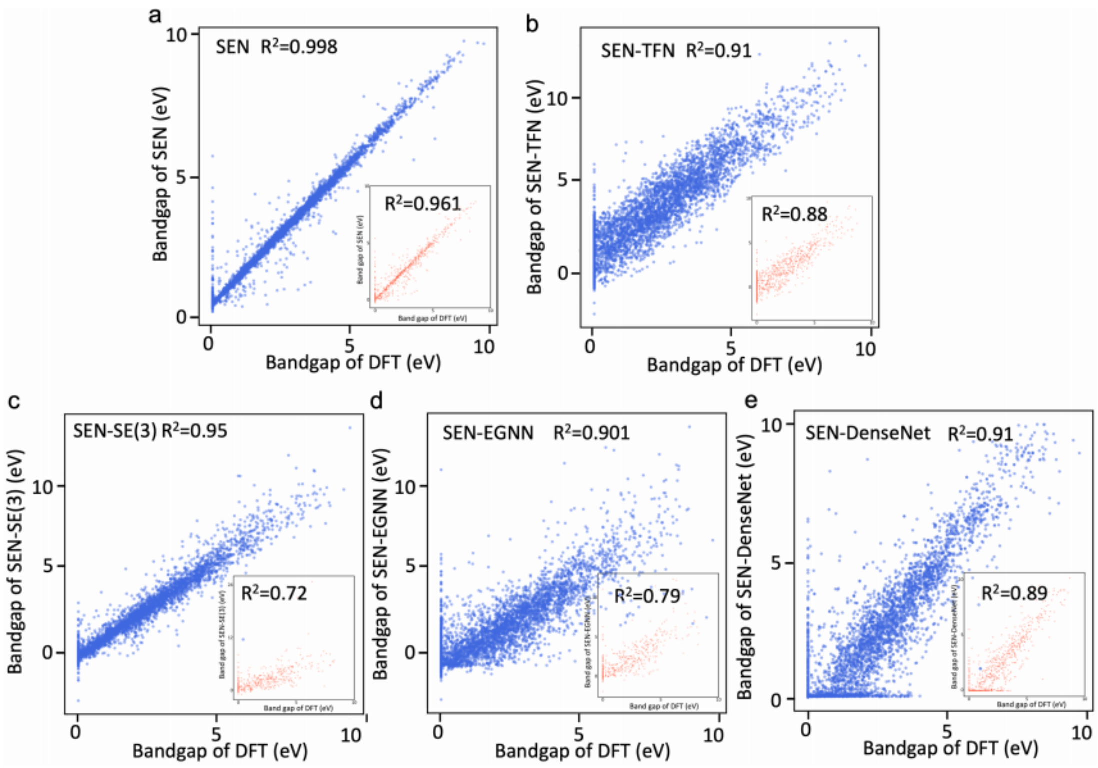

Supplementary Figure 22 The bandgap predictive results of (a) the SEN model, (b) the SEN-TFN model, (c) the SEN-SE(3)-Transformer model, (d) the SEN-EGNN model, and (e) the SEN-DenseNet model. The blue and red scatter plots are predictive results for training and testing datasets.

To enable qualitative comparison between our model and previous studies with the same data size, we have conducted additional calculations for predicting bandgap and formation energy with material datasets of different sizes (Supplementary Figure 23). Note that the bandgap calculation presented in the main text is performed using the dataset obtained by DFT method with the advanced HSE exchange-correlation functional, while the results presented in Supplementary Figure 22 are attained by the dataset with the traditional PBE exchange-correlation functional to be consistent with literature. The results clearly show the convergence trend of the test error as the training dataset increases for predicting the bandgap and formation energy. The performance of our SEN model on predicting both the bandgap and formation energy are superior to the existing algorithms due to the perception of crystal symmetry.

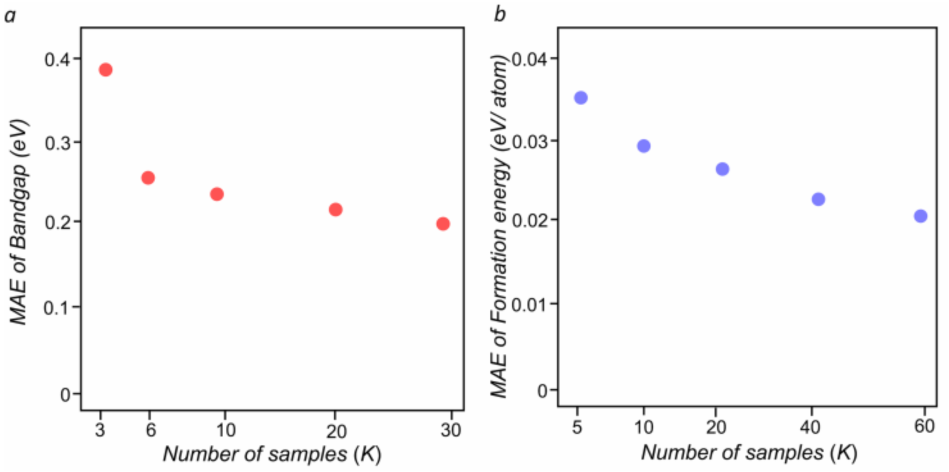

Supplementary Figure 23. Prediction performance of a bandgap and b formation energy via the SEN model with different number of samples.

In addition to the above performance comparison between the E(n)-based models and our SEN model, we performed more rational performance comparisons between the state-of-the-art graph-based models. Specifically, we have carefully tested the SEN model with the MatBench dataset, and thus enabled the fair comparison among the CGCNN[18], MEGNet[19], SchNet[22], and SEN models. For the models presented in Supplementary Table 5 but not encompassed in the MatBench website[25], we acquired the same datasets as the corresponding work, and examined them with the SEN model for fair comparisons.

We acquired the bandgap and formation energy datasets using the task function from Matbench Benchmark. The training and testing datasets of bandgap contain 84,873 and 21,222 materials respectively, while those of the formation energy contain 106,201 and 26,551 materials. The prediction performances of our SEN model for both the bandgap and formation energy were evaluated using exactly the same training and testing datasets as in the Matbench Benchmark.

The mean average errors (MAEs) of the testing datasets for predicting bandgap and formation energy with various models are summarized in Supplementary Table 5, wherein the MAEs of the CGCNN, MEGNet, and SchNet models are obtained from the MatBench website[25]. We have conducted extra tests on the CGCNN model to validate our implementation of the Matbench dataset. The resulted MAEs of 0.029 eV and 0.0368 eV/atom are consistent with those from the MatBench website. The mean prediction errors of our SEN model for bandgap and formation energy are 0.181 eV and 0.0161 eV/atom, achieving the highest prediction performance. The testing MAEs obtained by the SEN model for predicting bandgap are 39.1%, 6.2%, and 23.3% lower than those of the CGCNN, MEGNet, and SchNet models. Meanwhile, the testing MAEs

obtained by the SEN model for predicting formation energy are 52.6%, 36.1%, and 26.1% those of the CGCNN, MEGNet, and SchNet models.

Supplementary Table 5. Prediction performance comparison of four models on the MatBench.

**Number**	**Model**	**E_g(eV)**		**E_f(eV/atom)**	
		**84,873**	**21,222**	**106,201**	**26,551**
1	CGCNN[18]	0.297		0.0340	
1’	CGCNN (Our test)	0.29		0.0368	
2	MEGNet[19]	0.193		0.0252	
3	SchNet[22]	0.236		0.0218	
4	SEN	0.181		0.0161	

The $D(E_g)$ and $D(E_f)$ are the number of samples used for predicting E_g and E_f respectively.

The test results for the GATGNN[20] and AMDNet[21] models are not available in the MatBench website, and the codes of AMDNet model have not been distributed with their paper. In order to fairly compare with these models, we have performed additional tests on the SEN model adopting the same datasets as the above two models.

Supplementary Table 6. Prediction performance comparison of four models on the MatBench.

**Datasets (E_g / E_f)**	**Model**	**E_g (eV)**	**E_f(eV/atom)**
36720/60000	GATGNN[20]	0.31	0.048
	MEGNet[19]	0.33	0.028
	SEN	0.20	0.0184
22606/22606	AMDNet[21]	0.44	0.047
	MEGNet[19]	0.54	0.047
	SEN	0.039	0.0187

According to the relevant publications, the original tests on the GATGNN and MEGNet models share the same dataset, which comprises of crystal data from the Material Project database. The bandgap and formation energy datasets are download at GATGNN Github web including 36,720 and 60,000 materials respectively, wherein the crystals with finite bandgap dataset. The MAEs of testing datasets for predicting material properties obtained by the SEN, GATGNN, and MEGNet models using identical datasets are presented in Supplementary Table 8. The results suggest that the testing MAE obtained by the SEN model for predicting bandgap are35.5% and 39.4% lower than those of the GATGNN and MEGNet models. Meanwhile, 61.7% and 34.3% reductions of the testing MAEs are observed with the SEN model compared to the other

two models.

Regarding the comparison with the AMDNet model, we downloaded the data from the Materials Project database according to the selection criterion reported in literature, which includes 22,206 binary and ternary metal oxides. Consistent with previous study, the database was divided into a training set and a testing dataset containing 18,091 and 4,515 materials respectively. The prediction errors of the AMDNet and MEGNet models reported in the AMDNet paper as well as that of the SEN model are documented in Supplementary Table 6. The results indicate that the testing MAE obtained by the SEN model for predicting bandgap are 91.1% and 92.8% lower than the AMDNet and MEGNet models, while the testing MAEs for predicting formation energy are 60.2% lower than the two models. The prediction performance of our SEN model on this dataset is remarkably improved compared to that on the MatBench dataset, which probably can be ascribed to the elimination of metallic materials.

In both the unbiased test with the MatBench dataset and the fair tests with identical datasets, the prediction performance of the SEN model is outstanding compared to the other models. The outstanding performance of our model can be attributed to two key modifications in material simulation: learning symmetries and building chemical environments. On the one hand, the point group symmetry is a global material feature especially important for determining electronic structures. Our material capsule model encodes the crystal symmetry as a hidden (prior) descriptor to reflect the equivariant relationship between strengthens the mapping from material features to properties. On the other hand, the establishment of chemical environment can be viewed as a special hierarchical convolution operation that extracts joint structure-chemical patterns as higher-level material features. The atomic interactions can thus be taken into account in our predictive model, because the changes of weights associated with interacting features during training are correlated via the sharing of atomic environment.

Supplementary Note 9: The introduction for related models.

We first considered the distinctions between the SEN model and the existing ML studies on predicting the properties of crystal materials. Crystal graph convolutional neural networks (CGCNN, Xie and Grossman, 2018) presented a generalized crystal graph framework for property prediction on periodic crystal systems[18]. The CGCNN calculates correlations between atoms via convolution layer and the pooling layer is then used for producing an overall feature vector for the crystal. This method achieved accurate predictions (MAEs of 0.388 eV and 0.039 eV/atom) for bandgap and formation energy when 16k and 28k training data are used. MatErials graph network (MEGNet, Ong et al., 2019) proposed graph networks with additional global state

attributes to comprehensively reflect the underlying structure–property relationship[19]. A sequential update scheme of graph networks was designed for information flow among atoms, bonds, and global state. Excellent property predictions for bandgap and formation energy were accomplished, with the MAEs of 0.33 eV and 0.028 eV/ atom given 36.7k and 60k training data, respectively. GATGNN (Hu et al., 2020) proposed a deep graph neural network incorporating the l ocal and global attention mechanisms, which simultaneously learn the local relationship among neighboring atoms and the overall contribution of the atoms to the material's property[20]. The MAEs of 0.31 eV and 0.048 eV/atom are obtained for predicting bandgap and formation energy with 36.7k and 60k training data. Atom-motif dual graph neural network (AMDNet) (Vucetic and Yan et al., 2021) innovatively built structural features from the perspective of atomic clustering to better extract and transfer physical features[21]. Comparable MAEs (0.44 eV and 0.047 eV/ atom) were acquired for predicting bandgap and formation energy with 18k training data.

The above state-of-the-art graph models provide successful platforms for recognizing crystal features and predicting material properties, which are developed based on the conventional convolution network. Unfortunately, the translation invariance of the convolution operation and the indiscriminate superposition of the pooling layers inevitably lead to the incomplete extraction of structure features and the loss of rotational and reflection symmetry. These crystal graphic models thus suffer from the inability of perceiving crystal symmetry, even though the crystal symmetry is a global material feature especially important for determining electronic structures. Such drawback is illustrated by the large prediction errors of the MEGNet model for crystal systems with high symmetry. Our SEN model encodes the crystal symmetry as prior features of material capsules to reflect the equivariant relationship between basic material features, leading to a substantial reduction of effective feature dimensionality. Such feature purge process mitigates the overfitting problem and strengthens the mapping from material features to properties. This results in the substantial improvements of bandgap predictions for high symmetry crystal systems by the SEN model compared to those by the MEGNet model.

In ML models, material symmetry is typically perceived via the detection of equivariant patterns under spatial transformation within the E(3) group. Only a few studies have been conducted to expand the E(3) equivariance or E(3) invariance in material systems via redesigning the regular convolution filter. All of them considered molecular systems probably due to the simpler material symmetry compared to the crystal systems. SchNet (Sch¨utt et al., 2018) successfully described complex atomic interactions and predicted potential-energy surfaces, wherein the continuous-filter

convolutions are implemented to model the interaction term with the perception of the rotational invariance[22]. The rotational invariance is accomplished by using the relative distances rather than the atom coordination as input. A low MAE of 0.035eV/atom was achieved in the prediction of formation energy under 60k training data. In contrast to the equivariant networks that preserve the information of symmetry operations, the SchNet as a E(3) invariant network is not capable of describing the spatial relationship and the correlation between equivalent clusters within a material. Tensor field network (TFN) (Thomas et al., 2018) innovatively compiled a learnable spherical harmonics kernel which preserves the rotational equivariance but is expensive to compute[15]. Tests on the QM9 molecular database were conducted to recover molecular structures after randomly removing an atom from the original structures. However, the pairwise atomic distances rather than the atom coordination were used as input, which is naturally invariant in spherical harmonics representation. Both the material symmetry perception and accurate property prediction have not been demonstrated in this study. E(n)-equivariant graph neural networks (EGNNs) (Satorras et al., 2022) present a novel architecture to combine graph neural networks and coordinate embeddings, which achieves translation, rotation, and reflection equivariant (E(n)) as well as permutation equivariant[16]. This work also carried out tests on QM9 molecular data and achieved good performance. Nevertheless, as stated in their paper, coordinate embeddings and updating are not used in the molecular property prediction process, so it is impossible to verify whether the E(3) equivariance is achieved in this process. Recently, a novel E(3)-equivariant neural network (Batzner, and Kozinsky et al., 2022) based on neural equivariant interatomic potentials (NequIP) learned the interatomic potentials from ab-initio molecular dynamics simulations and accurately predicted the energy and forces of small organic molecules. Rotation and parity equivariances were encoded through a specific convolution layer incorporating learnable radial functions and spherical harmonics function[10].

The above E(3)-equivariant models mainly focusing on pure rotation symmetry in molecules are not sufficient for the describing the symmetry information of crystal materials. The crystal symmetry of a material may contain a variety of pattern equivariances under the spatial transformation of translation, rotation, reflection, mirror, and combinations of these operations with different reference points or planes. It is still possible to design complicated convolutional filter to realize the perception of crystal symmetry. Such model has not been proposed yet, and is likely to be even more computational demanding than the expensive spherical harmonic kernel.

The appearance of stacked capsule autoencoder (SCAE, Hinton et al., 2019), a novel E(n) equivariant model, sheds light on the other avenue to perceive crystal

symmetry[9]. The SCAE model uses different global transformations for every object as well as local transformations for each of their part capsules. The SCAE describes an image (object) as geometrical arrangements of parts, through intelligently discovering the important parts in an image and inferring their spatial relationships to the viewer (called the pose). To this end, the input is deconstructed into finite part capsules, and each capsule is composed of a six-dimensional pose, a presence variable, and a unique identity encompassing the remaining information. The pose matrix contains the translation, scale, rotation, reflection, mirror, and shear operations, as well as different combinations of these operations. Attention-based pooling is employed to predict the pose and existence of a particular part, with the constraint that this part is spatial transformation equivariance. The pose feature reflects not only the spatial relationship between the capsule and the object, but also the relative configurations between capsules.

Physically, the material properties mainly depend on the presence of important atomic clusters embedded in the crystal structure, the connectivity between clusters, and the structure-property relation determining the contribution of each cluster or cluster connection. The recognition of equivalent clusters arising from crystal symmetry within a material is crucial for predicting electronic structure. That is because the mapping from such clusters or relevant cluster interaction to material property is appropriately restricted to be identical, which is consistent with physical theory. Meanwhile, the recognition of almost equivalent clusters across different materials enhances the effective data size and improves the prediction accuracy. Without the equivalent cluster perception, the deep neural network is likely to suffer from the overfitting problem and converges to unrealistic solutions.

Within the above scenario, the crystal material fits into the concept of object in the SCAE model, while the various types of equivariant atomic clusters embedded in the crystal structure correspond to the part capsules. The implementation of capsule transformer on the material chemical environment generates a sufficient amount of part capsules representing critical local features, and enables the perception of complex equivariance by training the different pose vectors in different capsules. The perceptions of equivariances originating from translation, rotation, reflection, and mirror symmetry have been unambiguously demonstrated via the analysis of intermediate data in the SEN model. Besides, the preservation of spatial relationship between capsules facilitates the description of interactions between important clusters. In all, the SEN model based on the capsule transformer offers a flexible and efficient platform for recognizing the variety of complex symmetry and accurately predicting properties of crystal materials.

Supplementary Reference

[1] Loder, F., Kampf, A.P., Mannhart, J. et al. Institute of Solid State Physics. (2009).
[2] H Wondratschek, U. M. International tables for crystallography, Vol. A1. (2004).
[3] Cederberg, J. A course in modern geometries. Springer Science & Business Media. (2004).
[4] Donald E. Sands. Introduction to crystallography. (1975).
[5] Cohen, T. S., & Welling, M. Group equivariant convolutional networks. International Conference on Machine Learning. (2016).
[6] Fuchs, F. B., Worrall, D. E., Fischer, V. & Welling, M. Se(3)-transformers: 3d roto-translation equivariant attention networks. Adv. Neural Inf. Process. Syst. 33, 1970-1981. (2020).
[7] Finzi, M., Stanton, S., Izmailov, P. & Wilson, A. G. Generalizing convolutional neural networks for equivariance to lie groups on arbitrary continuous data. International Conference on Machine Learning. (2020).
[8] Jumper, J., Evans, R., Pritzel, A. et al. Highly accurate protein structure prediction with AlphaFold. Nature 596, 583–589. (2021).
[9] Kosiorek, A. R., Sabour, S., Teh, Y. W. & Hinton, G. E. Stacked capsule autoencoders. Adv. Neural Inf. Process. Syst. 32, (2019).
[10] Batzner, S., Musaelian, A., Sun, L. et al. E(3)-equivariant graph neural networks for data-efficient and accurate interatomic potentials. Nat. Commun. 13, 2453. (2022).
[11] Bruna, J., Zaremba, W., Szlam, A. & LeCun, Y. Spectral networks and locally connected networks on graphs. arXiv preprint arXiv:1312.6203. (2013)
[12] Defferrard, M., Bresson, X. & Vandergheynst, P. Convolutional neural networks on graphs with fast localized spectral filtering. Adv. Neural Inf. Process. Syst. 29, 3844–3852. (2016).
[13] Marcos, D., Volpi, M., Komodakis, N. & Tuia, D. Rotation equivariant vector field networks. In Proceedings of the IEEE International Conference on Computer Vision. (2017).
[14] Worrall, D. E., Garbin, S. J., Turmukhambetov, D. & Brostow, G. J. Harmonic networks: Deep translation and rotation equivariance. In Proceedings of International Conference on Computer Vision and Pattern Recognition. (2017).
[15] Thomas, N. et al. Tensor field networks: Rotation-and translation-equivariant neural networks for 3d point. arXiv preprint arXiv:1802.08219. (2018).
[16] Satorras, V. G., Hoogeboom, E. & Welling, M. E(n) equivariant graph neural networks. International conference on machine learning. PMLR. (2021).
[17] Kondor, R. & Trivedi, S. On the generalization of equivariance and convolution in neural networks to the action of compact groups. International Conference on Machine

Learning. PMLR. (2018).
[18] Xie, T. & Grossman, J. C. Crystal graph convolutional neural networks for an accurate and interpretable prediction of material properties. Phys. Rev. Lett. 120, 145301. (2018).
[19] Chen, C., Ye, W., Zuo, Y., Zheng, C. & Ong, S. P. Graph networks as a universal machine learning framework for molecules and crystals. Chem. Mater. 31, 3564–3572. (2019).
[20] Louis, S. Y. et al. Graph convolutional neural networks with global attention for improved materials property prediction. Phys. Chem. Chem. Phys. 22, 18141–18148. (2020).
[21] Banjade, H. R. et al. Structure motif–centric learning framework for inorganic crystalline systems. Sci. Adv. 7, 1754. (2021).
[22] Schütt, K. T., Gastegger, M., Tkatchenko, A., Müller, K. R. & Maurer, R. J. Unifying machine learning and quantum chemistry with a deep neural network for molecular wavefunctions. Nat. Commun. 10, 5024. (2019).
[23] Xu, D., Zhu, Y., Choy, C. B. et al. Scene graph generation by iterative message passing. Proceedings of the IEEE conference on computer vision and pattern recognition. (2017).
[24] Wolfram Hergert and R. Matthias Geilhufe. Group Theory in Solid State Physics and Photonics Problem Solving with Mathematica. (2018).
[25] Dunn, A., Wang, Q., Ganose, A., Dopp, D., Jain, A. Benchmarking Materials Property Prediction Methods: The Matbench Test Set and Automatminer Reference Algorithm. npj Computational Materials 6, 138 (2020)
